# Plasma polymerized nanoparticles effectively deliver dual siRNA and drug therapy in vivo

**DOI:** 10.1038/s41598-020-69591-x

**Published:** 2020-07-30

**Authors:** Praveesuda Michael, Yuen Ting Lam, Elysse C. Filipe, Richard P. Tan, Alex H. P. Chan, Bob S. L. Lee, Nicolas Feng, Juichien Hung, Thomas R. Cox, Miguel Santos, Steven G. Wise

**Affiliations:** 1 Department of Physiology, School of Medical Sciences, University of Sydney, Sydney, Australia; 2 Charles Perkins Centre, University of Sydney, Sydney, Australia; 3 Matrix and Metastasis Group, Garvan Institute of Medical Research, The Kinghorn Cancer Centre, UNSW, Sydney, Australia; 4 St. Vincent’s Clinical School, Faculty of Medicine, UNSW, Sydney, Australia; 5 Department of Cardiothoracic Surgery, Stanford University, Stanford, CA USA; 6 The University of Sydney Nano Institute (Sydney Nano), The University of Sydney, Sydney, Australia

**Keywords:** Biotechnology, Materials science, Nanoscience and technology, Physics

## Abstract

Multifunctional nanocarriers (MNCs) promise to improve therapeutic outcomes by combining multiple classes of molecules into a single nanostructure, enhancing active targeting of therapeutic agents and facilitating new combination therapies. However, nanocarrier platforms currently approved for clinical use can still only carry a single therapeutic agent. The complexity and escalating costs associated with the synthesis of more complex MNCs have been major technological roadblocks in the pathway for clinical translation. Here, we show that plasma polymerized nanoparticles (PPNs), synthesised in reactive gas discharges, can bind and effectively deliver multiple therapeutic cargo in a facile and cost-effective process compatible with up scaled commercial production. Delivery of siRNA against vascular endothelial growth factor (siVEGF) at extremely low concentrations (0.04 nM), significantly reduced VEGF expression in hard-to-transfect cells when compared with commercial platforms carrying higher siRNA doses (6.25 nM). PPNs carrying a combination of siVEGF and standard of care Paclitaxel (PPN-Dual) at reduced doses (< 100 µg/kg) synergistically modulated the microenvironment of orthotopic breast tumors in mice, and significantly reduced tumor growth. We propose PPNs as a new nanomaterial for delivery of therapeutics, which can be easily functionalised in any laboratory setting without the need for additional wet-chemistry and purification steps.

## Introduction

Nanotechnology has long promised to improve disease treatments by enhancing diagnosis, targeting, accumulation and controlled release of therapeutic agents^1–3^. Limitations in nanoparticle architecture, manufacture and functionalization have meant that these goals have so far been pursued independently. In cancer therapeutics for instance, all 15 clinically approved nanocarrier (NC) platforms have a single functionality, carrying one class of therapeutic cargo (e.g. Doxil, Abraxane, Myocet, ONIVYDE)^4^. Consequently, these nanotechnologies rely on inefficient passive targeting, resulting in less than 1% of the dose reaching the tumor^5^. Multifunctional nanocarriers (MNCs), carrying a combination of therapeutic cargo (e.g. drugs or genetic materials), imaging agents and targeting ligands, have improved therapeutic outcomes in animal models^4, 6–11^. MNCs have also been increasingly employed to co-deliver therapeutic cargo (e.g. peptides, drugs, siRNA, miRNA or DNA) to overcome drug resistance through synergistic effects^12–15^. However, these more complex multifunctional platforms, that either aim to improve active targeting or deliver a combination of therapeutics, have not progressed beyond clinical trials^4, 16, 17^. This lack of progress has been underpinned by both functional hurdles (e.g. due to cargo degradation, reduced endosomal escape and poor accumulation/retention at the diseased tissues), regulatory issues and technological roadblocks associated with the synthesis and functionalization of complex nanomaterials^18, 19^.

The complexity and costs associated with the synthesis of MNCs represent some of the most significant challenges in the pathway to translation^18^. The vast majority of NC materials are chemically inert or hydrophobic and therefore intrinsically unsuitable to tether molecular cargo. Combination of different classes of molecular cargo requires a versatile nanocarrier surface which is capable of binding molecules with a vast range of physical and chemical properties (molecular weight, charge, hydrophilicity/hydrophobicity). Furthermore, sequential addition of each new functionality often changes the surface properties of NCs (e.g. charge) at each binding step, reducing binding efficiency of subsequent cargo layers. Therefore, manufacturing complex MNCs which incorporate multiple therapeutic payloads and functionalities has thus far required multi-step, time-consuming and costly functionalization protocols. To circumvent the lack of functionality, the field relies heavily on wet-chemistry approaches to activate nanocarrier materials with chemical linkers to attach desired molecules^20, 21^. The incorporation of multiple classes of molecules with a variety of chemical structures, sizes and charge in a single NC requires optimization of different types of linkers and spacers to achieve maximum binding ability, cargo loading and optimal ratios between molecular cargoes. Further surface modification approaches have been applied to incorporate coatings, ligands or hydrophilic polymers that improve biocompatibility and half-life of the nano-construct or aim to control the release of the molecular cargo^4, 5, 10, 11, 21–24^. However, the addition of each new functionality represents extra synthesis, purification and quality control steps which compromises process reproducibility, reduces yield and significantly escalates the cost of production^25^. Such functionalization approaches represent significant manufacturing and scalability hurdles^19, 25, 26^ which hinder translation of MNCs. Cost/benefit considerations play a dominant role in the development of MNCs as the technology advances towards upscaled commercial production^18^. To reduce the cost of upscaled production and facilitate translation, multifunctional NCs should optimally be biocompatible, easy to synthesize and be able to bind multiple cargo in a simple one-step mixing process^11^. Single-step processes are also more suitable for good manufacturing practice (GMP) production and more efficient than multi-step processes where yield is lost in each synthesis or functionalization step.

**Figure 1:**
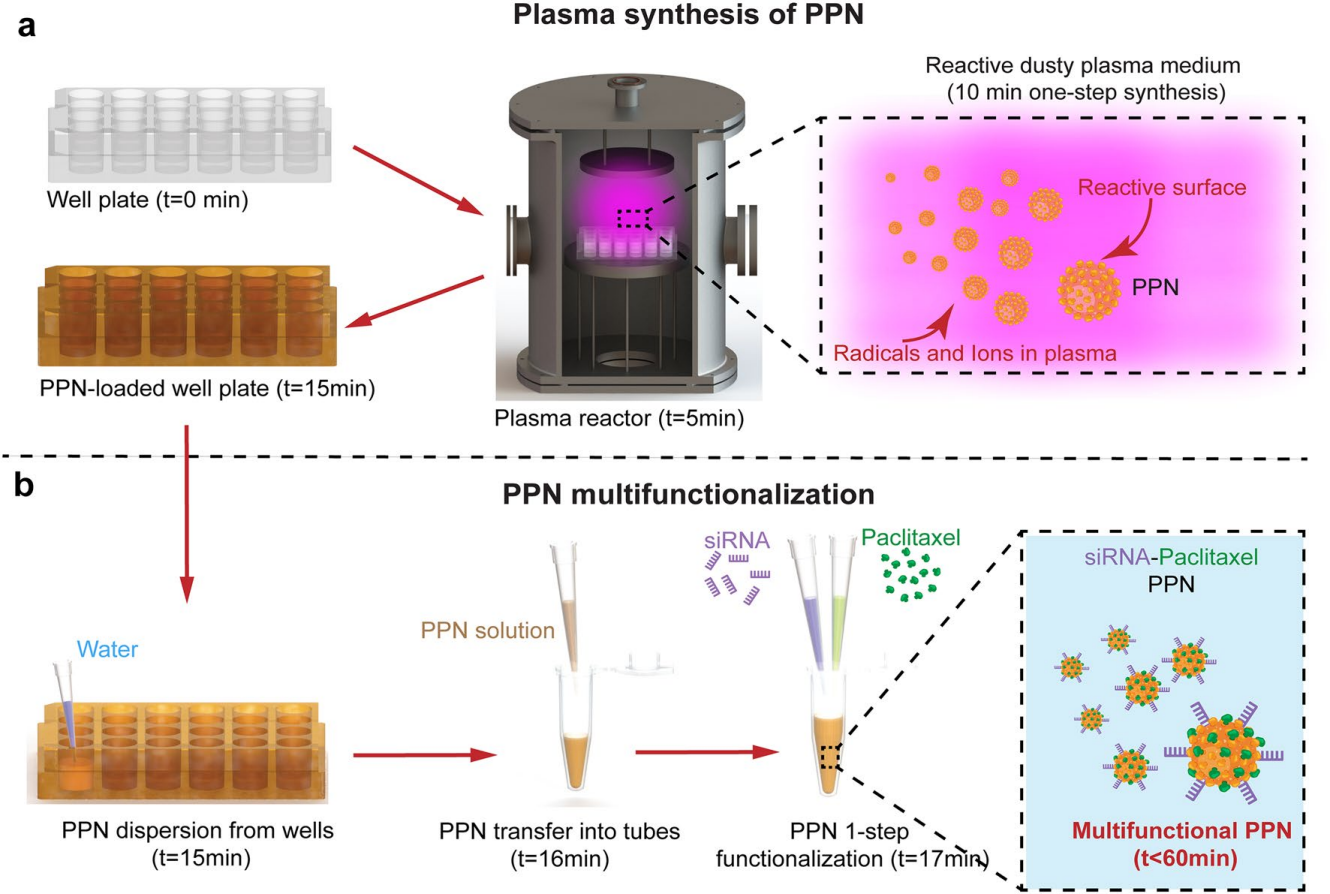
Synthesis and multifunctionalization of plasma polymerized nanoparticles (PPN). **(a)** Schematic depicting rapid and scalable synthesis process of PPN in a reactive plasma. PPN are formed by plasma polymerization via association of reactive species (ions and radicals) in the discharge bulk, yielding nanoparticles with high surface reactivity. **(b)** Schematic illustration of PPN multifunctionalization in a one-step process. Following synthesis, PPN can be readily dispersed in aqueous solution where binding with selected molecular cargo—exemplified here with Paclitaxel (PTX) and small interfering RNA (siRNA)—is performed without requiring additional linker chemistry or purification steps.

In this paper, we provide the first evidence that plasma polymerized nanoparticles (PPNs), a new class of nanocarriers^27^, can efficiently bind and carry multiple therapeutic cargo for in vitro and in vivo applications. PPNs are synthesised in plasma polymerization (PP) systems widely adopted for deposition of thin film coatings^28–30^ (Fig. 1a and Methods in Supplementary Information). In PP, a radiofrequency power source is employed to activate a gaseous mixture in a vacuum chamber. When using carbon precursors such as acetylene, reactive species (radicals and ions) are formed in the plasma bulk which eventually diffuse towards surfaces exposed to the plasma. These plasmas are often characterised by the formation of particulates in the chamber which compromise process efficiency. While long considered a by-product in plasma-based thin film coating, etching and energy applications^31, 32^, we recently demonstrated that plasma conditions can be optimized to produce high yields of stable PPNs^33^ with well-defined surface properties, favourable for biological applications^27^. Nanoparticle synthesis using this approach is a bottom-up dry process, readily scalable and cost-effective (Fig. 1a), making it compatible with commercial production^33^. Multi-functionalization of PPNs is achieved in a one-step process by simply mixing the nanoparticles with the molecular payload in aqueous solution, eliminating the need for linker intermediates (Fig. 1b). The binding versatility of PPNs is underpinned by a myriad of surface functional groups which are incorporated on the particles during the synthesis process. These reactive groups allow further modulation of the PPNs charge by simple modulation of the solution parameters during the binding process (pH, ionic strength and buffer type).

To demonstrate the utility of PPNs, we functionalized their surface with two classes of widely used therapeutic cargoes, siRNA against the vascular endothelial growth factor (siVEGF) and a standard of care frontline chemotherapy agent, Paclitaxel (PTX), and assessed their functionality in established in vitro and in vivo models.

## Results and discussion

**Figure 2:**
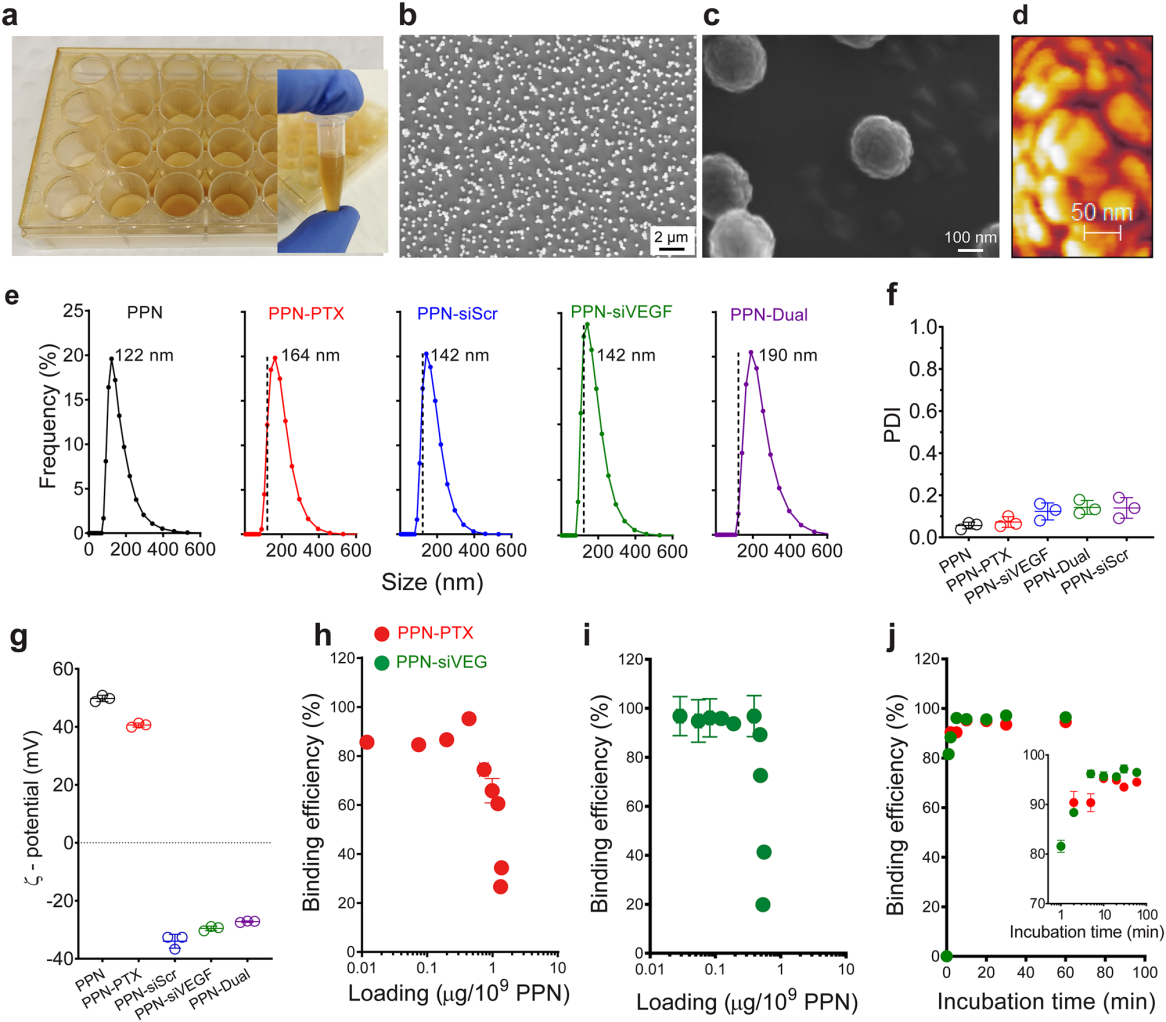
Characterization and binding properties of plasma polymerized nanoparticles (PPN). **(a)** The synthesis process in a plasma reactor allows collection of PPN in well plates for direct dispersion of nanoparticle aliquots in solution and transfer into tubes. **(b)**, **(c)** Scanning electron microscopy (SEM) images of as-synthesized PPN collected on a silicon wafer placed inside a well. **(d)** High-resolution atomic force microscopy (AFM) phase scan details the surface topography of a single PPN, which is formed by aggregation of smaller nanoclusters. **(e)** Hydrodynamic size distribution, **(f)** polydispersity index (PDI) and **(g)** zeta potential (ζ-potential) of unfunctionalized and PPN bound to paclitaxel (PPN-PTX), siRNA-scrambled (PPN-siScr), siRNA against the vascular endothelial growth factor (PPN-siVEGF) and both PTX and siRNA-VEGF (PPN-Dual). **(h)**, **(i)** Binding efficiency vs loading capacity and **(j)** vs incubation time of PPN to PTX and siRNA-VEGF.

To produce PPNs, a radiofrequency discharge is sustained in a vacuum chamber at a constant power and working pressure (100 W and 150 mTorr respectively) using a reactive mixture of acetylene (C_2_H_2_), nitrogen (N_2_) and argon (Ar). This process produces a uniform distribution of PPNs which can be collected and concentrated directly from the plasma bulk into well-shaped collectors^33^, such as tissue culture well plates (Fig. 2a). Since the plasma is also a sterilization source, PPNs can be dispersed in aqueous solution directly from the collectors into tubes (Fig. 2a insert), allowing rapid functionalization with molecular cargo without the need for further post-processing or purification steps. The reactive surface of PPNs eliminates the need for linker-chemistry and the functionalization process can be easily adopted in any laboratory setting. The efficient synthesis process uniformly yields nanoparticles at a mass rate of 85 mg/h^33^. PPNs have a long shelf-life and can be stored at room temperature up to at least 16 months after plasma synthesis for subsequent functionalization with molecular cargo^27^. Plasma polymerized materials differ from conventional polymers, in that they do not retain the same homogeneous, repeating structure. The structure is characterized by a highly branched, heterogeneous and random network of structural units. In the case of PPNs, ionization and dissociation of C_2_H_2_ and N_2_ creates ions and radical species that polymerize into reactive CN:H amorphous nanoclusters^27^. The carbonaceous nanoclusters assemble into spherical-like nanoparticles (Fig. 2b–d) which are dragged outside the plasma by ion drag and thermophoretic forces^27, 33^. Chemical characterization of PPNs was consistent with our previous reports on PP-derived materials^33, 34^, confirming incorporation of elemental carbon and nitrogen into the nanoclusters followed by surface restructuring due to oxidation (Figure S1a) and the presence of amine and carboxyl functional moieties (Figure S1b). These surface functional groups improve surface hydrophilicity^35, 36^, significantly increase surface reactivity and can be readily protonated (or de-protonated) in solution by changing pH ^37^, allowing fine tuning of surface charge and stability in solution ^27^. The versatile surface properties of PPNs facilitate robust tethering of clinically relevant molecules of various sizes, charge and chemical structures using a simple one-step incubation in aqueous solution^27^. PPNs allow bypass of post functionalization and purification steps required by multi-step wet-chemistry protocols, hence reducing yield loss and facilitating upscaled manufacturing.

To explore the potential of PPNs to bind and deliver multiple functional cargo we bound paclitaxel (PPN-PTX), siRNA targeting VEGF (PPN-siVEGF) or a combination of paclitaxel and siVEGF (PPN-Dual). The rationale for this choice of combination therapy was based on several physico-chemical and biological considerations that allowed us to independently test the binding and delivery of two major classes of therapeutic cargo in a single structure while demonstrating the therapeutic potential of PPNs in well established in vitro and in vivo settings. Furthermore, combinations of PTX with siRNA therapies have been proposed as promising combination therapy in cancer treatment^38, 39^. All binding experiments were conducted at room temperature by simple incubation and mixing of as-synthesized PPNs dispersed in physiological pH distilled water (directly from wells—Fig. 1b and Fig. 2a) with reconstituted aqueous solutions of the molecular cargo. The binding of PPNs to PTX and siVEGF was first evaluated with dynamic light scattering to measure changes in the particles hydrodynamic size, mobility and surface charge. Dispersion of unfunctionalized PPNs in water at physiological pH yielded a monodisperse population of positively charged particles with a median hydrodynamic size 122 ± 24 nm (Fig. 2e), polydispersive index (PDI) 0.06 ± 0.01 (Fig. 2f) and Zeta potential 50 ± 1 mV (Fig. 2g). The positive surface charge in water at physiological pH is likely driven by protonation of surface functional groups, e.g. amines, which improve solution stability through inter-particle electrostatic repulsion^40^. PPNs increased in size following functionalization with all molecular cargo (142 ± 19 nm for PPN-siVEGF, 164 ± 31 nm for PPN-PTX and 190 ± 48 nm for PPN-Dual) but changes to PDI were not significant between groups (Fig. 2f). Binding of negatively charged RNA inverted the surface charge of all PPN groups carrying siRNA in water (− 30 ± 1 mV for PPN-siVEGF and − 27 ± 1 mV for PPN-Dual), while PPN-PTX remained positively charged (41 ± 1 mV). The loading capacity and binding efficiency of PPNs is influenced by the molecular weight of the cargo, the molecular footprint on the nanoparticle surface and the PPN/molecule concentration ratio in solution^27^. We determined the loading capacity, binding efficiency (i.e. fraction of the initial cargo bound to nanoparticles when added to a PPN aqueous solution) and binding kinetics of PPN to molecular cargo using fluorescently labeled siVEGF (Cy5) and PTX-OregonGreen-488 and established optimal incubation parameters to maximize binding efficiency and cargo loading. This optimization was based on the PPN/molecule concentration ratio in solution previously optimized to achieve a theoretical monolayer of cargo on the nanoparticles surface^27^. The binding efficiency of PPN to PTX-OregonGreen-488 (*M*_w_ = 1,319 Da) reached a maximum of 95% (Fig. 2h), corresponding to a loading capacity of 0.4 ± 0.02 μg/10^9^ particles (2.0 × 10^5^ PTX molecules per particle). The loading capacity could be further increased to 1.4 ± 0.02 μg/10^9^ particles (6.4 × 10^5^ PTX molecules per particle) but at a lower binding efficiency of 34% due to excess PTX in solution. The maximum binding efficiency of PPN to si-VEGF-Cy5 (*M*_w_ = 13.5 kDa) was 97%, representing a mass loading capacity of 0.4 ± 0.03 μg/10^9^ particles or 1.8 × 10^4^ siVEGF molecules per particle (Fig. 2i). Surface saturation occurred at 0.55 ± 0.01 μg/10^9^ particles (2.5 × 10^4^ siVEGF molecules per particle) corresponding to a binding efficiency of 41%. During preparation of dual functionalized PPN formulations (PPN-Dual) we found that PTX was partially desorbed from the surface of PPN with increasing concentration of siVEGF in solution (Figure S2a), potentially due to the Vroman effect^41^. Therefore, PPN-Dual formulations were prepared by sequential binding of siVEGF first followed by PTX. No elution of siVEGF from PPN was found following binding of PTX in excess (Figure S2b). Negatively charged siRNA is robustly tethered to the positively charged surface of PPN via electrostatic interaction. We expect adsorption of paclitaxel, a lipophilic molecule, to be facilitated by C-H aliphatic compounds (Figure S1b) present on the surface of PPN. We then measured the binding kinetics of PPNs to siVEGF and PTX (Fig. 2j) to establish the incubation time at optimal cargo concentrations in solution (i.e. the point of maximum binding efficiency previously determined). PPN bound 90% of all PTX molecules present in solution in the first 2 min of incubation while maximum loading capacity was achieved in the first 10 min of incubation. The binding of siVEGF molecules occurred at a faster rate, with full loading capacity observed with only 5 min of incubation. Together, these results show that as-synthesized PPN can carry dense molecular payloads at a high binding efficiency (very close to 100%) without addition of chemical linkers, further purification or sample processing, hence greatly simplifying NC multifunctionalization.

**Figure 3:**
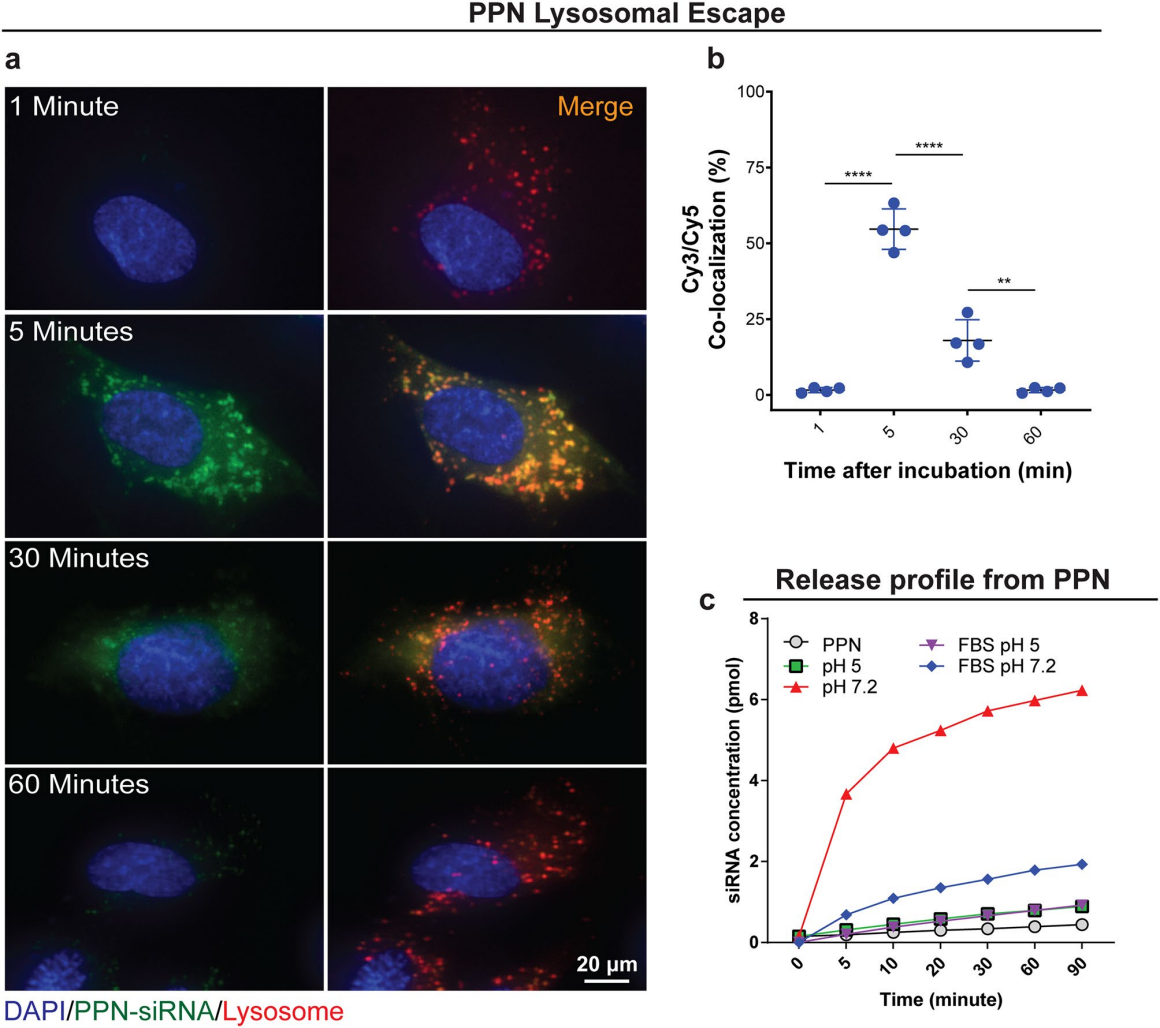
Rapid internalization and lysosomal escape of plasma polymerized nanoparticles (PPN) in human coronary endothelial cells (hCAEC). **(a)** Confocal microscopy images of hCAEC at different timepoints following incubation with PPN functionalized with Cy5-tagged siRNA against the endothelial growth factor (PPN-siVEGF-Cy5). Lysosomes/endosomes were stained with LysoTracker Deep Red. Only lysosomes/endosomes (red) are visible 1-min following incubation with nanoparticles. Internalization of PPN-siVEGF-Cy5 (green) by endocytosis is observed at 5 min, as suggested by the yellow signal inside the lysosomes upon merging of both fluorescent channels. By 30 min, virtually all particles escape lysosome compartments (shown again in red). Release of siVEGF-Cy5 from PPN is suggested by a decrease and diffusion of the green fluorescence throughout the cell. **(b)** Co-localization of siVEGF(Cy5) and Lysosomes/endosomes (LysoTracker Deep Red) in hCAEC at various time points following incubation of cells with PPN-siVEGF. **(c)** Release profile of siRNA-siVEGF from PPN in citrate buffer with or without foetal bovine serum (FBS) at pH 5 and pH 7.2. Data are presented as mean ± s.d. (n = 4). ***p < 0.005 and ****p < 0.0001 versus PPN-siSCR. Experiments were repeated three times.

Suitable vector systems rely on nanocarriers that enhance siRNA cellular uptake, can circumvent the cellular recycling pathways and ameliorate cytosolic siRNA release. Cationic lipid-based nanoparticles (LNP) have progressed farthest in siRNA delivery for cancer applications^42^. Cationic LNPs interact electrostatically with negatively charged nucleic acids, forming positively charged heterogeneous lipid complexes (lipoplexes) that efficiently encapsulate siRNA molecules^43^. Lipoplexes improve siRNA cellular uptake in vitro via endocytosis, while further membrane fusion with the endosome walls facilitates siRNA release into the cytosol^44^. However, the cytotoxicity of cationic lipoplexes, due to their interference with membrane function and integrity of the cell and subcellular compartments, remains a problematic drawback^45^. Furthermore, interaction of cationic lipoplexes with negatively charged proteins in the blood compromises siRNA bioavailability and accumulation in target tissues, hampering efficacy. Following internalization, it is crucial that NCs remain stable and rapidly escape from endosomes/lysosomes in order to release siRNA into the cytosol where therapeutic effects occur^46^. Confinement of NCs in membrane bounded vesicles, including lysosomes, can prevent their release into the cytosol, lead to degradation of biological cargo and potentially result in cellular excretion^47^. A significant proportion (up to 70%) of siRNA delivered via LNPs is lost through exocytosis^48^. The relatively rapid lysosomal escape of carriers designed to influence cell function is therefore crucial to their efficacy^49^. To determine if PPNs escape endosome/lysosome compartmentalization, PPN-siVEGF (Cy5) were incubated with human coronary artery endothelial cells (hCAECs) for up to 60 min (Fig. 3a). Here, LysoTracker Deep Red, a red-fluorescent dye for labelling acidic organelles (i.e. lysosomes and endosomes), and PPN-siVEGF (Cy5) were utilized to observe intracellular trafficking. Co-localization of PPN-siVEGF(Cy5) and lysosomes (55 ± 7% co-localization) was detected 5 min after incubation (Fig. 3a, b), reflected in the abundant yellow staining in the merged image. This confirms a rapid uptake of PPNs and their entrapment in the early phase of internalization. We note that the processes of endosomal-lysosomal encapsulation, starting from biomolecule internalization, to the development of early endosomal vesicles, to late endosomal vesicles, have been reported to occur as soon as 5 to 15 min^50^. Xia et al.^51^ has also demonstrated that 30 nm polystyrene nanoparticles entered endosomes within 10 min, showing that cellular uptake through the endosomal-lysosome system can be rapidly achieved. By 30 min, a dispersion of siRNA-Cy5 (green) is clearly observed throughout the cytosol and outside the lysosomes (18 ± 7% co-localization), suggesting that PPN-siVEGF (Cy5) promptly escapes endosomal compartmentalization.

A significant decrease in green fluorescence was observed by 60 min, suggesting release of siRNA from the PPN surface and subsequent dispersion into the cytosol after endosomal escape. In contrast, a lipofectamine-siVEGF(Cy-5) group was not visible within hCAECs in the first 60 min (Figure S3), a result consistent with previous reports^52^, suggesting a significantly slower internalization mechanism compared with PPN. A possible candidate mechanism for PPN endosomal escape is known as “proton sponge effect” and commonly observed for cationic nanocarriers^53^. The “proton sponge effect” results from acidification of the endosomes following cellular uptake of nanocarriers, causing an osmotic imbalance that triggers significant water uptake, vesicle swell and ultimately membrane rupture. Other proposed mechanisms suggest that siRNA release could occur inside the endosomes and subsequently be injected into the cytoplasm via transient pores within the membrane^54^. While these two mechanisms may not be necessarily mutually exclusive, we conducted a Calcein assay to determine whether disruption of the endosomal membrane occurs following PPN internalization. Calcein is a membrane impermeable fluorescent dye which self-quenches when concentrated in the acidic environments inside endosomes^55^. A diffuse and intense fluorescence occurs if the membrane is disrupted and Calcein escapes into the cytosol. We added Calcein bound to PPN (PPN-Calcein) to hCAEC and performed confocal microscopy to visualise PPN-Calcein intracellular trafficking over time (Figure S4). Results are consistent with those obtained with PPN-siRNA. Punctate, low intensity and localised distribution of Calcein is observed inside endosomes within the first 10 min following incubation with the cells. After 30 min, diffuse and high intensity green fluorescence is observed throughout the cells, suggesting endosomal escape and supporting the hypothesis that molecular cargo bound to PPN rapidly escape endosomal entrapment via possible membrane disruption.

While the most common strategy to enable the cytosolic release from lipid- or conventional polymeric- based nanocarriers is to functionalise the nanocarrier surface with cationic polymers or peptides^56^, PPNs did not require additional surface modification to achieve rapid cytosolic release once internalized into the cells. We evaluated the release kinetics of siRNA from PPNs in different pH buffers to further elucidate the release of siRNA observed following PPN lysosomal escape. We observed minimal release of siRNA from PPNs (Fig. 3c) in conditions mimicking the acidic environment (pH5) of endosomes/lysosomes irrespective of the presence of serum. A stronger interaction between PPN and negatively charged siRNA is expected at lower pH due to protonation of surface amine groups (Figure S1b), which drives an increase in positive net charge around the PPN surface. A burst release of siRNA was observed in the first 5 min following incubation of the nanoparticle system in sodium citrate buffer in simulated physiological pH (7.2), in the absence of serum. At progressively higher pH, de-protonation of carboxylic-acid groups lower the zeta-potential of PPN ^27^, weakening the interaction between PPNs and siRNA. Interestingly, slower release rates were observed in sodium citrate buffer (pH 7.2) in the presence of serum, with 3 times less siRNA eluted from PPNs after 90 min compared with the same pH conditions without serum.

**Figure 4:**
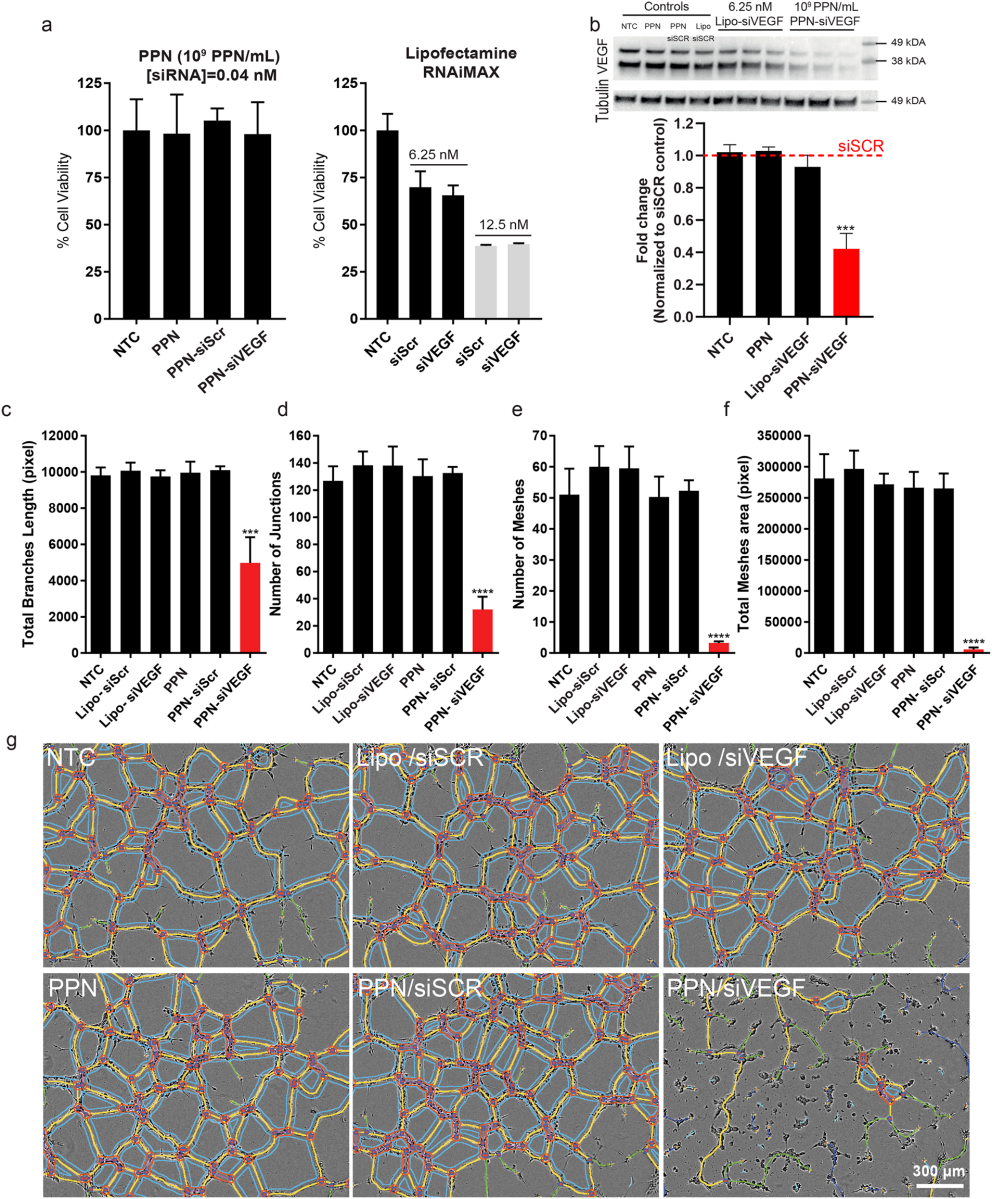
Enhanced transfection efficiency in hard-to-transfect human coronary endothelial cells (hCAEC) with plasma polymerized nanoparticles (PPN). **(a)** hCAECs viability upon delivery of siRNA against the endothelial growth factor (siVEGF) and siRNA scrambled (siScr) with PPN (10^9^ PPN/mL) and Lipofectamine RNAiMAX (6.25 nM and 12.5 nM). **(b)** Levels of siVEGF expression in hCAEC quantified by Western Blot following 24 h treatments. A cropped version is shown here due to space constraints. The full and uncropped blot is shown in Figure S5. Delivery of siVEGF with PPNs carrying siRNA concentrations as low as 0.04 nM (10^9^ PPN/mL) significantly reduced VEGF expression compared with Lipofectamine RNAiMAX, even at significantly higher siRNA concentrations (6.25 nM). **(c)**–**(g)** Delivery of functional siVEGF via PPNs significantly reduced the total branch length, number of junctions, number of meshes and total mesh area of tubules formed by hCAEC in a Matrigel tubulogenesis assay. Data are presented as mean ± s.d. (n = 4). ***p < 0.001 and ****p < 0.0001 versus PPN-siSCR. Experiments were repeated three times.

To evaluate whether siRNA delivered by PPNs remains functional following rapid cell internalization and endosomal release, we delivered PPN-siVEGF in difficult to transfect primary endothelial cells (hCAECs) and assessed VEGF knock-down. Primary endothelial cells are notoriously difficult to transfect^57^ with commercially available NCs platforms failing either due to low transfection rates or high cytotoxicity. Unfunctionalized PPNs and PPN-siSCR were employed as negative control groups, while a commercial lipid-based nanocarrier platform for delivery of siRNA (Lipo RNAiMAX) was utilized as a positive control (Lipo-siVEGF). We first evaluated the cytotoxicity of PPNs and Lipo RNAiMAX carrying control scrambled siRNA (siSCR) and siVEGF to account for any decrease in protein expression caused by toxicity effects. Cell viability was unaffected upon delivery of all PPN formulations tested at concentrations up to 10^9^ PPN/mL (Fig. 4a). Delivery of Lipo RNAiMAX at siRNA concentrations recommended by the manufacturer (12.5 nM) significantly reduced hCAEC viability to 38.70 ± 0.43% (lipo-siSCR; p < 0.0001) and 39.63 ± 0.40% (lipo-siVEGF; p < 0.0001) compared to cells with no treatment. These results are consistent with previous studies employing lipid-based NCs, demonstrating their toxicity in primary cells^58, 59^. A reduction in the concentration of Lipo RNAiMAX (carrying 6.25 nM of siRNA) improved viability levels (69.8 ± 6.93%; p < 0.0001 for Lipo-siSCR and 65.5 ± 4.2% p < 0.0001 Lipo-siVEGF), but hCAEC viability was still significantly lower than any PPN formulation tested. The expression of VEGF protein in hCAECs transfected with PPN-siVEGF (10^9^ PPN/mL, corresponding to an siRNA concentration of 0.04 nM) 24 h following incubation was significantly reduced to 42.15 ± 9.61% (p = 0.0001) when normalized to PPN-siSCR control (Fig. 4b and Figure S5). Cells treated with Lipo-siVEGF had no discernible effect with VEGF expression reduced to 92.99 ± 7.25% (non-significant against Lipo-siSCR control). We note that this level of reduction in protein expression is superior to that of the currently available siRNA-nanoparticle platforms, given the extremely low concentration of siVEGF (0.04 nM) used in this study^60^.

The significant decrease in VEGF expression in cells transfected with PPN-siVEGF translated to impaired tubule formation (Fig. 4c–g) in an established Matrigel assay ^61^. The total branch length (Fig. 4c), number of junctions (Fig. 4d), number of meshes (Fig. 4e), and total mesh area (Fig. 4f) were all strikingly reduced (p < 0.0001), further demonstrating the effectiveness of siVEGF delivered with PPNs in hard-to-transfect primary cells. All Lipofectamine RNAiMAX formulations were equivalent to non-treatment controls. Overall, these results suggest that PPNs are a powerful transfection vehicle, effectively delivering functional siRNA in hard-to-transfect primary cells with no detectable cytotoxicity at concentrations tested.

**Figure 5:**
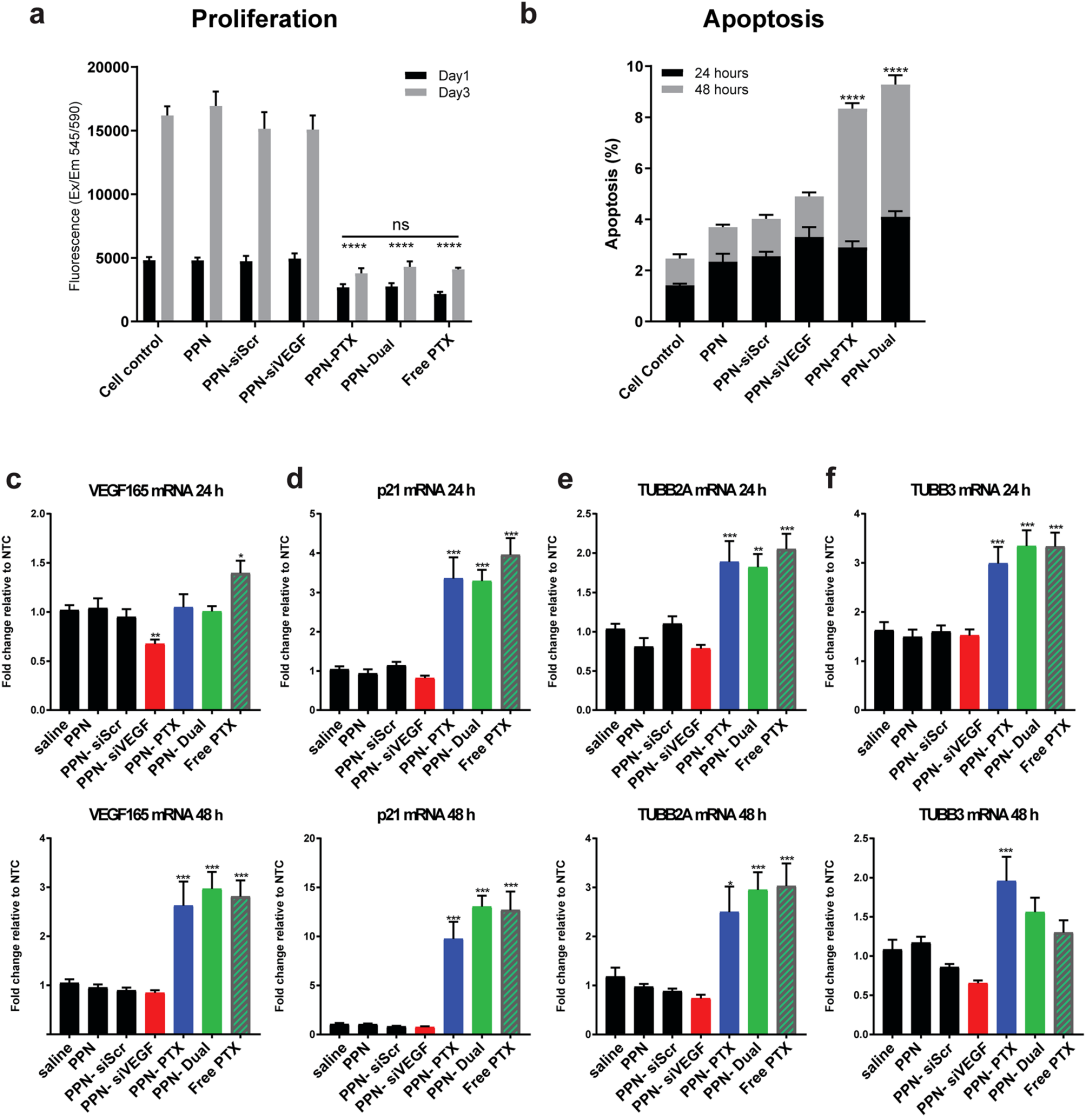
Delivery of dual functionalized plasma polymerized nanoparticles (PPN) with siRNA against the vascular endothelial growth factor (siVEGF) and paclitaxel (PTX) in MCF7 breast cancer cells. **(a)** The proliferation of MCF7 was significantly inhibited upon delivery of PPN functionalized with PTX only (PPN-PTX) and both PTX and siVEGF (PPN-dual) after 1- and 3-days post-treatments. **(b)** Consistent with the anti-proliferative results, a significant increase in apoptotic cells was observed in the PPN-PTX and PPN-dual formulations. **(c)** Expression levels of VEGF_165_, **(d)** p21, **(e)** TUBB2A and **(f)** TUBB3 24 h and 48 h post treatments determined by RT-qPCR. VEGF_165_ expression was significantly suppressed by PPN-siVEGF at 24 h but elevated by PPN-PTX, PPN-dual and free PTX at 48 h **(c)**. Consistently, expression levels of p21 **(d)**, TUBB2A **(e)** and TUBB 3 **(f)** were significantly increased by free PTX, PPN-PTX or PPN-dual formulations. All Data are presented as mean ± s.d. (n = 4). * p < 0.05, ** p < 0.01, *** p < 0.001 versus saline control.

We next sought to establish the potential of PPNs to bind and deliver two classes of molecules, a frontline standard-of-care chemotherapy drug (PTX) and siVEGF, in a different in vitro setting. Drug and siRNA combination therapies using LNP carriers have been proposed as promising for treatment of cancer^62^. However, multifunctionalization of LNPs rely on the same multi-step, wet chemistry protocols discussed above, which limits clinical translation. Here, we functionalized PPNs with both PTX and siVEGF using the same one-step incubation and mixing process in aqueous solution (Fig. 1b), prior to administration onto MCF-7 breast cancer cells. We combined PTX and siVEGF to exemplify the multifunctionality of PPNs and show the feasibility of delivering multiple payloads using a synthesis and functionalization process compatible with clinical translation. We carried out alamarBlue and Incucyte live cell assays to investigate cytotoxicity and determine the anti-proliferative effects of unfunctionalized PPNs, PPN-siSCR (0.15 µg/mL), PPN-siVEGF (0.15 µg/mL), PPN-PTX (0.25 µg/mL), and PPN-Dual (PTX 0.125 µg/mL, siVEGF 0.075 µg/mL) in MCF7 cells. Untreated cells, and free PTX were used as negative and positive controls respectively. Cell proliferation was significantly inhibited (Fig. 5a and Figure S6) when treated with PPN-PTX (55.6 ± 4.59% reduction compared to untreated cells at 24 h and 23.4 ± 2.17% at 72 h), and PPN-Dual (57.01 ± 4.78% at 24 h and 26.48 ± 2.30% at 72 h). Free PTX was comparable with a reduction to 44.98 ± 2.94% at 24 h and 25.28 ± 0.76% at 72 h. Unfunctionalized PPN, PPN-siSCR, PPN-siVEGF groups had no discernable effects 24- and 72-h post-incubation with cells. Cell apoptosis analysis using Annexin V/PI cell staining (Fig. 5b) was consistent with the alamarBlue assay, showing a significant increase in apoptotic cells for samples treated with PPN-PTX and PPN-Dual groups. Representative images of cell density and morphology after 24- and 72-h post-treatments are illustrated in Figure S6.

We further evaluated the efficiency of single and dual functionalized PPN formulations in MCF7 cells by quantifying the expression levels of VEGF_165_ (the most abundant splice isoform of VEGF in human cells) and genes known to be critical in regulating tumor cell proliferation and survival, including anti-tumor p21, β-tubulin 2A (TUBB2A) and β-tubulin 3 (TUBB3) using RT-qPCR. PPN-siVEGF significantly reduced the expression of VEGF_165_ (Fig. 5c) compared to saline control after 24 h (0.68-fold decrease, p < 0.01). However, treatment with PPN-Dual had no significant effect on VEGF expression levels compared to saline control. After 48 h, VEGF suppression was attenuated in PPN-siVEGF while its expression was substantially increased in PPN-PTX, PPN-Dual and free PTX groups. VEGF expression was also increased by treatment of free PTX (1.39-fold at 24 h; 2.81-fold at 48 h), indicating that PTX induction of VEGF was not an artefact of PPN. The increase of VEGF expression in MCF7 cells was consistent with previous reports that PTX induces. VEGF expression through the production of reactive oxygen species in cancerous cell lines^63^. It is possible that co-delivery of PTX masks the effect of siVEGF on MCF7 in vitro. On the other hand, treatment with PPN-PTX and PPN-Dual induced a threefold increase in p21 expression and, approximately twofold and threefold increase in tubulin subclasses TUBB2A and TUBB3, respectively, in MCF7 cells compared to saline control (Fig. 5d–f). The expressions of p21, TUBB2A and TUBB3 of PPN-PTX and PPN-Dual were not significantly different compared to free PTX, indicating that PTX efficacy is not reduced when complexed with PPN or complexed together with siVEGF. Interestingly, PPN-PTX induced TUBB3 expression was significantly higher than free PTX at 48 h (P = 0.02), suggesting that the effect of PTX on gene expression may be partially prolonged by complexing with PPN. These outcomes were consistent with the known action of PTX, which inhibits cell division by disrupting the mitotic spindle through the stabilization of microtubules^64^. After 48 h, the expression of p21, TUBB2A and TUBB3 remained elevated in PPN-PTX, PPN-Dual and free PTX groups. Together, these results indicate that PPN delivers functional siRNA targeting of VEGF and induces cell cycle inhibition by PTX action in vitro.

**Figure 6:**
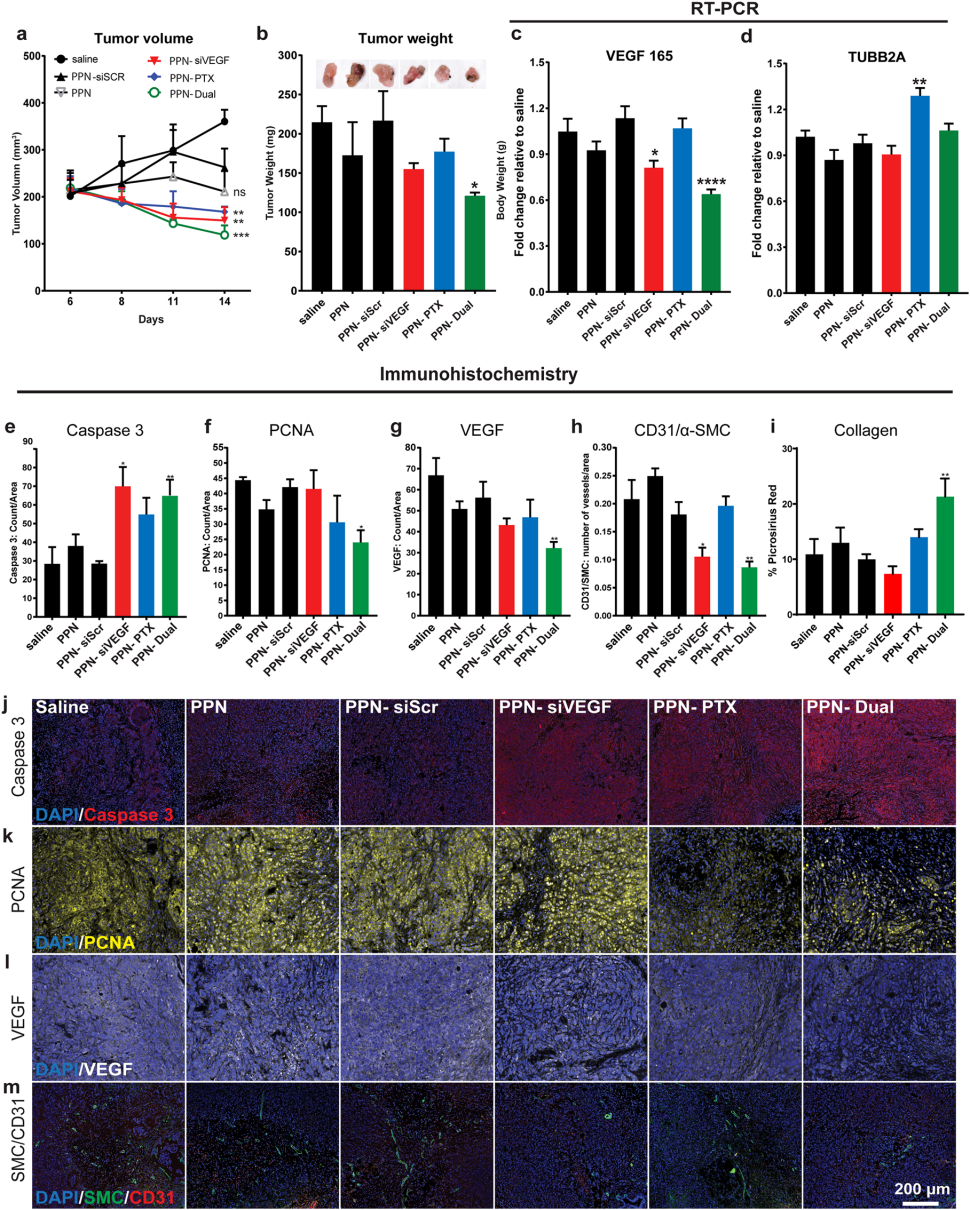
In vivo performance of plasma polymerized nanoparticle (PPN) formulations in a mouse orthotopic breast cancer model. **(a)**, **(b)** Tumors treated with PPNs dual functionalized with siRNA against the endothelial growth factor and paclitaxel (PPN-Dual) were threefold smaller compared to the vehicle control (saline), regressing 40% in volume **(a)** and 43% in weight **(b)** following administration of 3 doses spanning over 14 days. **(c)**, **(d)** Quantitative RT-PCR showed a significant reduction in the expression of VEGF_165_ **(c)** in tumors treated with PPN-siVEGF and PPN-Dual and a significant elevation of TUBB2A **(d)** for PPN-PTX compared to the saline control. **(e)**–**(m)** Tumor immunohistochemistry staining analysis of Caspase 3, proliferating cell nuclear antigen (PCNA), VEGF and ratio of platelet endothelial cell adhesion molecule-1 (CD31), α-smooth muscle actin (α-SMA) and collagen deposition. Results show that PPN-Dual formulations uniformly modulate tumor micro-environment, significantly increasing cell apoptosis (**e**, **j**) and collagen deposition (**i**) while inhibiting cell proliferation (**f**, **k**), VEGF expression (**g**, **l**) and angiogenesis (**h**, **m**). All data is presented as mean ± s.d. (n = 4). **p < 0.01, and *p < 0.05 versus saline control group. Scale bar = 200 µm.

We next assessed the efficiency of multifunctional PPN formulations in vivo in orthotopically grown MCF-7 primary breast tumors (Figure S7), a well-established tumor model for screening NCs carrying chemodrugs^65, 66^. Tumors were allowed to grow 6 days up to 200 mm^3^, a tumor volume significantly higher than typically adopted in inhibition studies (50 mm^3^), hence representing a more challenging setting. Furthermore, we delivered significantly smaller doses of PTX (< 100 µg/kg) and siRNA (< 75 µg/kg) compared to those typically adopted by conventional classes of NCs (~ mg/kg for both PTX and siRNA in similar models). We evaluated tumor progression in vehicle (saline), PPN, PPN-siSCR, PPN-siVEGF, PPN-PTX, and PPN-Dual (Fig. 6a, b) treated animals. The animal survival rate was 100% with all mice reaching the pre-determined 14-day endpoint. Delivery of PPN formulations including PTX, siRNA or the dual combination resulted in a significant regression in tumor volume compared to vehicle following administration of 3 doses over 7 days. Tumors treated with PPN-Dual formulations were threefold smaller (p < 0.001) compared to vehicle at day 14, regressing 40% (p < 0.005) and 43% (p < 0.05) from their initial volume and weight respectively. The body weight of the mice remained unchanged throughout the period of the study for all groups (Figure S8). We carried out RT-qPCR analysis in explanted tumors to evaluate anti-angiogenic effects of siVEGF delivered by PPN-siVEGF and PPN-Dual formulations by quantifying VEGF_165_ expression. The cytotoxic effects of PPN-PTX and PPN-Dual were examined by quantifying TUBB2A^67, 68^. As shown in Fig. 6c, VEGF mRNA expression was suppressed in both PPN-siVEGF and PPN-Dual groups, with a 0.8-fold and 0.6-fold reduction compared to vehicle, respectively. While VEGF inhibition was lost for PPN-Dual in MCF7 cells in vitro (Fig. 5c), VEGF expression was effectively suppressed by dual functionalized PPN carrying siVEGF and PTX in vivo. MCF7 cells generated estrogen-dependent solid tumors consisting of peri- and intra-tumoral vessels in vivo. Gene silencing mediated by PPN-Dual may target, not only MCF7 cells, but also other VEGF-expressing cells that interact with MCF7 within the tumor microenvironment. Concurrently, PPN-PTX treatment led to a 1.3-fold increase in TUBB2A expression compared to saline control 48 h following the last injection (Fig. 6d).

We further conducted a comprehensive suite of immunohistochemistry (IHC) staining (Fig. 6e–m) to evaluate changes to the tumor microenvironment including cell proliferation, apoptosis, protein expression, angiogenesis and formation of extracellular matrix. We selected caspase3 as a marker for apoptotic cells, and proliferating cell nuclear antigen (PCNA) as a marker for cell proliferation. Caspase3, a cysteine protease enzyme, plays a key role in initiating apoptotic DNA fragmentation upon cellular damage^69^. Significantly higher levels of caspase3 were found in tumors treated with PPN-siVEGF (2.47-fold increase, p < 0.05) and PPN-Dual (2.29-fold increase, p < 0.01) when compared to vehicle control (Fig. 6e). Representative images of tumor sections in all treatment groups stained with caspase 3 (represented in red) are illustrated in Fig. 6j. Levels of PCNA (Fig. 6f, k) significantly decreased in tumors treated with PPN-Dual formulations (57% reduction, p < 0.05). Tumors were also stained for pro-angiogenic VEGF (Fig. 6g, l), platelet endothelial cell adhesion molecule-1 (CD31) and α-smooth muscle actin (α-SMA) to assess VEGF expression (Fig. 6g, l) as well as vasculature distribution and density in tumors (Fig. 6h, m). Consistent with RT-PCR results (Fig. 6c), IHC quantification of VEGF protein was significantly lower in tumors treated with PPN-Dual formulations, which showed a 1.57-fold reduction (p < 0.05) compared with vehicle. Downregulation of VEGF was concomitant with a significant reduction in tumor neovascularization. Tumors receiving vehicle control displayed a 2.4-fold (p < 0.01) higher number of vessels compared with those treated with PPN-Dual formulations (Fig. 6h, m). A significant reduction (twofold) in vessel number per area (p < 0.01 compared to vehicle) was also found for tumors treated with PPN-siVEGF. Lastly, clinical response to chemotherapy and the resulting debulking of breast tumours is often accompanied with a detectable deposition of fibrotic extracellular matrix, and in particular fibrillar collagen^70, 71^. The localized cell death caused by effective chemotherapy treatment triggers a wound healing response in the tissue which underpins this significant collagen deposition. Clinically used tumour regression grading systems (Mandard, Dowrak) frequently quote fibrosis as a positive indicator of disease regression, with increasing levels of fibrosis generally being associated with enhanced disease regression and decreased likelihood of recurrence^72^. Our results show that the dual therapy was effective in generating a significant fibrotic response in the tumours (Fig. 6i and Figure S9), with significantly higher collagen density within the tumour compared to control samples, (1.96-fold increase, p < 0.005), in line with the decreased tumour volume seen in Fig. 6a. Interestingly, cell proliferation, apoptosis, VEGF expression, collagen production and neovascularization were all modulated in tumours that received dual PPN-Dual formulations. This modulation demonstrates a potential synergy between siVEGF and PTX, showing that therapeutic cargo carried by PPN remains functional upon local delivery.

## Conclusion

The formation of PPN in plasma reactors has been previously described as an industrial contaminant but their application for biology has only been recently suggested. This work demonstrates for the first time the therapeutic potential and functionality of PPN for in vitro and in vivo applications. We show that PPNs carrying siRNA are internalized by cells and rapidly escape endosomal compartmentalization, preserving the molecular cargo with minimal degradation. When delivered to hard-to-transfect cells (hCAEC), PPNs carrying small doses of siVEGF (0.04 nM) significantly reduced VEGF expression with no cytotoxicity compared with commercially available lipid-based platforms carrying higher siRNA doses (6.25 nM). Dual functionalized PPN formulations carrying significantly reduced doses of PTX (< 100 µg/kg) and siRNA (< 75 µg/kg) compared with currently available NCs modulated the tumor microenvironment in an orthotopic breast cancer model resulting in significantly smaller tumors, increased cell apoptosis, decreased proliferation and tumor vascularization. In this work, all therapeutic cargo was loaded onto PPNs using a single and rapid incubation step in aqueous solution, eliminating conventional wet-chemistry protocols and purification steps. Together, we have demonstrated the promise of a new nanocarrier that overcomes many of the limitations of commercials platforms, providing facile, cost effective multifunctionalisation without compromising bioactivity. Outperforming currently available agents, PPN provides a simple and robust strategy for gene delivery and combination therapy.

## Supplementary information

Supplementary Information.

## Abbreviations

AFM: Atomic force microscopy
CD31: Platelet endothelial cell adhesion molecule (PECAM-1)
GMP: Good manufacturing practice
hCAECs: Human coronary artery endothelial cells
IHC: Immunohistochemistry
LNP: Lipid-based nanocarriers
MNCs: Multifunctional nanocarriers
mRNA: Messenger ribonucleic acid
*M*_w_: Molecular weight
NC: Nanocarrier
p21: Cyclin-dependent kinase inhibitor 1
PCNA: Proliferating cell nuclear antigen
PDI: Polydisperse index
PP: Plasma polymerization
PPN: Plasma polymerized nanoparticles
PTX: Paclitaxel
RT-qPCR: Real-time polymerase chain reaction
SCR: Scrambled
SEM: Scanning electron microscopy
siRNA: Small interfering ribonucleic acid
TUBB2A: Tubulin beta-2a
VEGF: Vascular endothelial growth factor
α-SMA: α-Smooth muscle actin
