# Supplementary material for: Plasma polymerized nanoparticles effectively deliver dual siRNA and drug therapy in vivo
Source: Sci Rep. 2020 Jul 30;10:12836. doi: 10.1038/s41598-020-69591-x (PMC7393381; doi:10.1038/s41598-020-69591-x)
Supplement: Supplementary file 1 — Supplementary Information. [file 41598_2020_69591_MOESM1_ESM.docx]

Supporting Information

Plasma Polymerized Nanoparticles Effectively Deliver Dual siRNA and Drug Therapy in vivo

Praveesuda Michael^a,b^, Yuen Ting Lam^a,b^, Elysse C. Filipe^c,d^, Richard Tan^a,b^, Alex HP Chan^e^, Bob SL Lee^a,b^, Nicolas Feng^a,b^, Juichien Hung^a,b^, Thomas R. Cox^c,d^, Miguel Santos^a,b,#,*^ and Steven G. Wise^a,b,#,*^

^a^School of Medical Sciences, Department of Physiology, University of Sydney, Australia

^b^Charles Perkins Centre, University of Sydney, Australia

^c^Matrix and Metastasis Group, Garvan Institute of Medical Research and The Kinghorn Cancer Centre, UNSW Sydney, Australia

^d^St Vincent's Clinical School, Faculty of Medicine, UNSW Sydney, NSW 2010, Australia.

^e^Department of Cardiothoracic Surgery, Stanford University, California USA.

#Correspondence should be addressed to

Miguel Santos (email: [miguel.correiadossantos@sydney.edu.au](mailto:miguel.correiadossantos@sydney.edu.auhri.org.au))

or

Steven Wise (email: [steven.wise@sydney.edu.au](mailto:steven.wise@sydney.edu.au))

## **Materials and methods**

**PPN synthesis and collection**

PPN were synthesized and collected in a cylindrical stainless steel capacitively coupled radiofrequency reactor as previous described ^1, 2^. A radiofrequency (13.52 MHz) discharge was generated at a pressure of 150 mTorr by ionization of a gaseous mixture of argon, nitrogen and acetylene. The flow rate of each gas was maintained constant throughout PPN synthesis. PPN were collected in 24-well polystyrene plates (Corning) directly from the plasma and subsequently dispersed in RT-PCR grade water (Life Technology) directly from the wells.

### **Scanning and transmission electron microscopy**

SEM was performed with a Zeiss ULTRA Plus or a Zeiss Sigma HD FEG scanning electron microscope at acceleration voltages ranging 3 – 10 kV and a working distance between 3 – 12 mm.

### **Laser-light scattering for hydrodynamic size, concentration and zeta potential measurements**

The hydrodynamic size distribution and concentration of PPN in RT-PCR Grade water was measured in a NanoSight NS300 laser light scattering system as previously described ^2^. For each measurement, samples were introduced into the analysis chamber and allowed to flow at a constant flow rate and temperature (24˚ C). The trajectories of PPN in the chamber were visualized and tracked using a nanoparticle tracking and analysis system (NanoSight NTA 3.0). Data for each set of samples resulted from the continuous tracking (60 seconds periods) of the PPN trajectories and subsequent statistical analysis of five independent measurements. The zeta potential of PPN was measured within a pH range of 2 – 10 in disposable folded capillary cells (Malvern, DTS1070) using a Zetasizer Nano ZS (Malvern Instruments, Germany). Each sample was measured at a constant temperature of 24˚ C and the zeta potential was obtained from the average of three independent series of 11 measurements each. The quality of the measurements was monitored in real time and the data was analyzed using standard procedures using the software provided by the manufacturer.

## **X-Ray photoelectron spectroscopy**

Chemical characterization of PPN was studied by means of X-ray photoelectron spectroscopy (XPS) using a K-Alpha X-ray XPS system ^2^. The system was operated at a constant pressure of ~10^-8^ Torr USING a X-Ray Spot size of 200 µm. Survey spectra of the samples were scanned in the energy range of 100 eV – 1400 eV using an energy step of 1 eV and an energy pass of 30 eV. High resolution scans for C1s, N1s and O1s were recorded with an energy step of 0.03 eV and an energy pass of 23 eV. Peak analysis was performed using Avantage software. The atomic fraction of these elements in the particles was determined by calculating the integrated areas of the C1s, N1s and O1s peaks and assuming that they sum to 100% (i.e. neglecting hydrogen and traces of other elements).

**Fourier transform spectroscopy in attenuated total reflectance mode (FTIR)**

Infrared spectra were recorded by means of Fourier transform spectroscopy in attenuated total reflectance mode (FTIR-ATR) using Bruker Alpha spectrometer (MA, USA)^2^. Each spectrum resulted by averaging a total of 64 scans at a spectral resolution of 4 cm^-1^ in the wavenumber range of 4000 cm^-1^ – 500 cm^-1^. Spectral subtraction and baseline were applied to eliminate background signal from the underlying substrate.

## **In vitro siVEGF release study**

PPN (10^9^ particles/mL) were conjugated to siRNA-Cy3 (Sigma, SIC005) in RT-PCR Grade water (Life Technology, 4387936) for 20 minutes at room temperature. PPN alone were used as a control group. All samples were then washed 3 times via centrifugation at 16,100g for 5 minutes and reconstituted with either sodium citrate buffer; pH5 (to simulate physiological pH in endosomes/lysosomes) or sodium citrate buffer; pH7.2 (to simulate physiological pH in the cytosol). At pre-determined time points (5, 10, 20, 30, 60 and 90 minutes), the samples were centrifuged at 16,100g for 5 minutes and the supernatants collected for analysis.

**Calcein conjugated PPN lysosomal escape**

Human coronary artery endothelial cells (HCAECs) – (CELL APPLICATIONS, INC., cat. no. 300-05a) – were seeded at 80% confluence on 24-well plates. Twenty-four hours after cell seeding, cells were stained with LysoTracker Deep Red (L12492, ThermoFisher Scientific) at the working concentration of 75 nM and NucBlue Live ReadyProbes Reagent according to the manufacturer’s instructions for 1 hour. Calcein conjugated PPN (100nm) at concentrations of 2 µM were added into each well. Non-treatment cell control (NTC) and calcein only were used as control groups. The images were taken under a fluorescent microscope in 3 different channels (Hoechst 33342 Ex/Em: 360/460, PPN-calcein Ex/Em:490/525, LysoTracker Ex/Em: 647/668) at pre-determined time points (5, 10, 30 and 60 minutes).

## **siRNA conjugated PPN lysosomal escape**

Human coronary artery endothelial cells (HCAECs) were seeded at 70% confluence on a Lab-Tek glass chamber slide (154534, Lab-Tek). Twenty-four hours after cell seeding, cells were stained with LysoTracker Deep Red (L12492, ThermoFisher Scientific) according to the manufacturer’s instructions at the working concentration of 75 nM for 1 hour. At pre-determined time points (1, 5, 30 and 60 minutes), siRNA conjugated PPN at concentrations of 10^9^ particle/mL (100 nm), and 2.5x10^8^ particle/mL (200 nm) were added into each well. Non-treatment cell control (NTC), PPN only, and lipofectamine-Encapsulated siRNA prepared as per manufacturer’s instructions (13778100, ThermoFisher Scientific) were used as control groups. The cells were then fixed with 3.7% paraformaldehyde for 10 minutes and washed 3 times with PBS prior to cell nucleus staining using NucBlue Live ReadyProbes Reagent. The cells were then washed twice in PBS and cover-slipped using ProLong Gold Antifade Mountant (P10144, Life Technologies). The images were taken within 24 hours under a fluorescent microscope in 3 different channels (Hoechst 33342 Ex/Em: 360/460, PPN Ex/Em:490/525, LysoTracker Ex/Em: 647/668).

## **Western blot assay and endothelial cell tubule formation**

PPN (10^9^ nanoparticles/mL) were conjugated to siRNA-VEGF (GCAACAUCACCAUGCAGAUtt) (Millenium Science, J-003500-08-0005) or siRNA Universal Negative Control (siSCR) (Sigma-Aldrich, SIC001) at a concentration of 40 pmol and applied to hCAECs. After 48-hours of treatment, cells with siVEGF and siSCR conjugated PPN (and respective control samples) were lysed using Mammalian Cell Lysis Kit (Sigma-Aldrich, MCL1). The total protein concentration was determined using QuantiPro BCA Assay Kit (Sigma-Aldrich, QPBCA) and 10µg of total protein per sample was loaded into a Blot 4-12% BIS-Tris Plus Gel (Life Technology, NW04120) and ran on BioRad Gel system at 120V for 1 hour. The protein gel was then transferred to a iBlot2 Nitrocellulose membrane (ThermoFisher, IB23001) using the iBlot2 Gel Transfer Device (ThermoFisher, IB21001) as per manufacturer’s instructions. The membrane was blocked with 5% milk powder in TBS-T with agitation for 1 hour. Staining for VEGFA protein was performed with an anti-VEGFA primary antibody (abcam, ab46154) at a dilution of 1:2000 in 2% milk powder in TBS-T overnight at 4 °C. After three, 5-minute washes with TBS-T, the membrane was incubated with goat anti-rabbit-HRP secondary antibody (Santa Cruz Biotech., sc2030) at a 1:5000 dilution in 2% milk powder in TBS-T, for 2 hours at room temperature. After three, 5-minute washes in TBS-T, the signal was developed using Luminata Forte Western HRP substrate (Millipore, WBLUF0100) and serial images were acquired by a BioRad Gel Doc XR system for analysis (BioRad Software). The membrane was then stripped and re-probed in an identical fashion with anti-α-tubulin conjugated to HRP (Abcam, ab40742) at a dilution of 1:5000 in 2% milk in TBS-T.

For the tubulogenesis assay, cells treated with siVEGF and siScrambled conjugated PPN, and respective controls, were trypsinized after 24 hours and re-plated onto a Matrigel (Corning, 354248) coated 96 well plate at a density of 10000 cells/0.32 cm^2^. Cells were then monitored over a period of 16 hours and time-lapse images were captured using IncuCyte Zoom Live Cell Imager (Essen Bioscience). Four representative images and analysis of the capillary network at 5 hours’ post seeding, for each replicate well was chosen for analysis. Analysis was performed using the angiogenesis analyzer plugin for Image J and included total branch length, number of junctions, number of meshes, and total mesh area.

## **Cell viability and proliferation assays**

MCF7 were cultured on 96 well-plates at 60 % confluency 24 hours prior to the study. Culture growth medium was removed and replaced with 10^9^ particles/mL (total volume 100 µL per well) of PPN, PPN-siSCR (siSCR 0.015µg/0.32cm^2^), PPN-siVEGF (siVEGF 0.015µg/0.32cm^2^), PPN-PTX (PTX 0.025µg/0.32cm^2^), PPN-PTX-siVEGF or PPN-Dual (PTX 0.0125µg/0.32cm^2^, siVEGF 0.0075 µg/0.32cm^2^) or PTX (PTX 0.25µg/0.32cm^2^) suspended in normal growth media. Non-treatment control (NTC) was used as a negative control. Cells were incubated with PPN at 37 °C, 5% CO_2_. At pre-determined time points (Day 1 and 3), PPN and the control samples were removed, 90 µL of fresh growth media and 10 µL of alamarBlue reagent were added into each well. After 2 hours incubation with the reagent, alamarBlue fluorescence (EX/EM: 530-560/590 nm) was then measured via CLARIOstar microplate reader. Relative cell viability and proliferation percentage were calculated and normalized against NTC.

## **Cell apoptosis assay**

MCF7 (1x10^5^ cells/well) were plated into a 12-well plate 24 hours prior to the study. The cells were then treated with 3×10^9^ particles/mL (total volume 100 µL per well) of PPN, PPN-siSCR (siSCR 0.045µg/4cm^2^), PPN-siVEGF (siVEGF 0.045µg/4cm^2^), PPN-PTX (PTX 0.075µg/4cm^2^), PPN-PTX-siVEGF or PPN-Dual (PTX 0.0375µg/4cm^2^, siVEGF 0.0225 µg/4cm^2^) or PTX (PTX 0.25µg/0.32cm^2^). Non-treatment controls (NTC) were utilized as a control group. After 24 hours incubation, the cells were harvested and stained with Annexin V-FITC/propidium iodide (PI) staining as per the manufacturer’s instructions. Briefly, all samples were incubated for 15 minutes at 37 °C with 5 µL Annexin V-FITC, 5 µL PI diluted in 490 µL 1x binding buffers (50 mM HEPES, 700 mM CaCl_2_, pH 7.4). Apoptotic and necrotic cells were immediately analysed via flow cytometry.

## **Real-time quantitative PCR (qPCR)**

Total RNA was extracted from MCF7 cells or tumor tissue using TriReagent (93289, Sigma). Extracted total RNA was reverse transcribed with a High-capacity cDNA reverse transcription kit (4368814, ThermoFisher). The expression levels of VEGF, TUBB2A, TUBB3 and p21 were examined. GAPDH and beta-actin were used as housekeeping genes for normalization. PCR conditions were as follows: 3 min at 95^o^C; 30 sec at 95^o^C, 30 sec at 60^o^C and 30 sec at 72^o^C (×40 cycles). Each run included negative reaction controls. Expression levels were calculated by the relative quantification method (∆∆Ct). RT-qPCR was performed in triplicate for each sample.

## **In vivo subcutaneous breast cancer model**

SCID mice (female, 5-6 weeks of age, body weight between 16-19 g) were selected for the subcutaneous breast cancer study under approval from the SLHD Animal Welfare Committee (AWC); protocol number: 2015/041C. All experiments were performed in accordance with relevant guidelines and regulations. MCF-7 cells (2x10^7^) cells in 120 µL of PBS mixed with 60 µL of Matrigel were injected subcutaneously into the mammary region of the mice. The tumor size was accessed using a Vernier caliper. The tumor volume was then calculated using the standard formula:

$V=\frac{(W^{2}\times L)}{2}$ ; where V= tumor volume (mm^2^), W = tumor width, L = tumor length

Once the tumor volume had reached approximately 200 mm^2^, the mice were randomized into 6 different groups (n = 4). The treatments; saline, PPN, PPN-siSCR, PPN-siVEGF, PPN-Paclitaxel, PPN-Dual with the dose of 100 µg/kg PTX and 75 µg/kg siRNA; were then administered via peritumoral injection on day 6, 8, 11, 14, respectively. The therapeutic efficacy was evaluated post-injection by monitoring tumor volume and body weight for the period of 14 days. At the end of the study, all the mice were sacrificed 48 hours post-final-injection, tumor tissues were harvested, and weighed to obtain the final tumor weight. Tumor tissues were then collected for qPCR and histological study.

## **Real-time quantitative PCR (qPCR)**

Total RNA was extracted from MCF7 cells or tumor tissue using TriReagent (93289, Sigma). Extracted total RNA was reverse transcribed with a High-capacity cDNA reverse transcription kit (4368814, ThermoFisher). The expression levels of VEGF, TUBB2A, TUBB3 and p21 were examined. GAPDH and beta-actin were used as housekeeping genes for normalization. PCR conditions were as follows: 3 min at 95^o^C; 30 sec at 95^o^C, 30 sec at 60^o^C and 30 sec at 72^o^C (×40 cycles). Each run included negative reaction controls. Expression levels were calculated by the relative quantification method (∆∆Ct). RT-qPCR was performed in triplicate for each sample.

## **Histological studies**

Tumour samples were fixed overnight in paraformaldehyde (4 %) at room temperature. Samples were dehydrated through an ethanol gradient and embedded in paraffin and sectioned at 5 μm. For immunohistochemistry staining, sections were deparaffinized and stained with antibodies against SM α-actin (Sigma-Aldrich, a5691, 1:500) for smooth muscle cells, CD31 (Abcam, ab28364, 1:200) for endothelial cells, PCNA (Abcam, ab29, 1:500) for proliferative cells, Caspase 3 (Abcam, ab, 1:200) for apoptotic cells and VEGF (Abcam, ab52917, 1:200).

## **Data analysis**

All data is displayed as mean ± standard deviation (SD) with three repeats in all tested samples (n = 3). Ordinary one-way analysis of variance (ANOVA) followed by Dunnett’s multiple comparison test was applied for multiple comparisons of three or more group means. P < 0.05 was considered statistically significant. ****, ***, **, and * display P < 0.0001, P < 0.001, P <0.01, and P < 0.05, respectively.

**
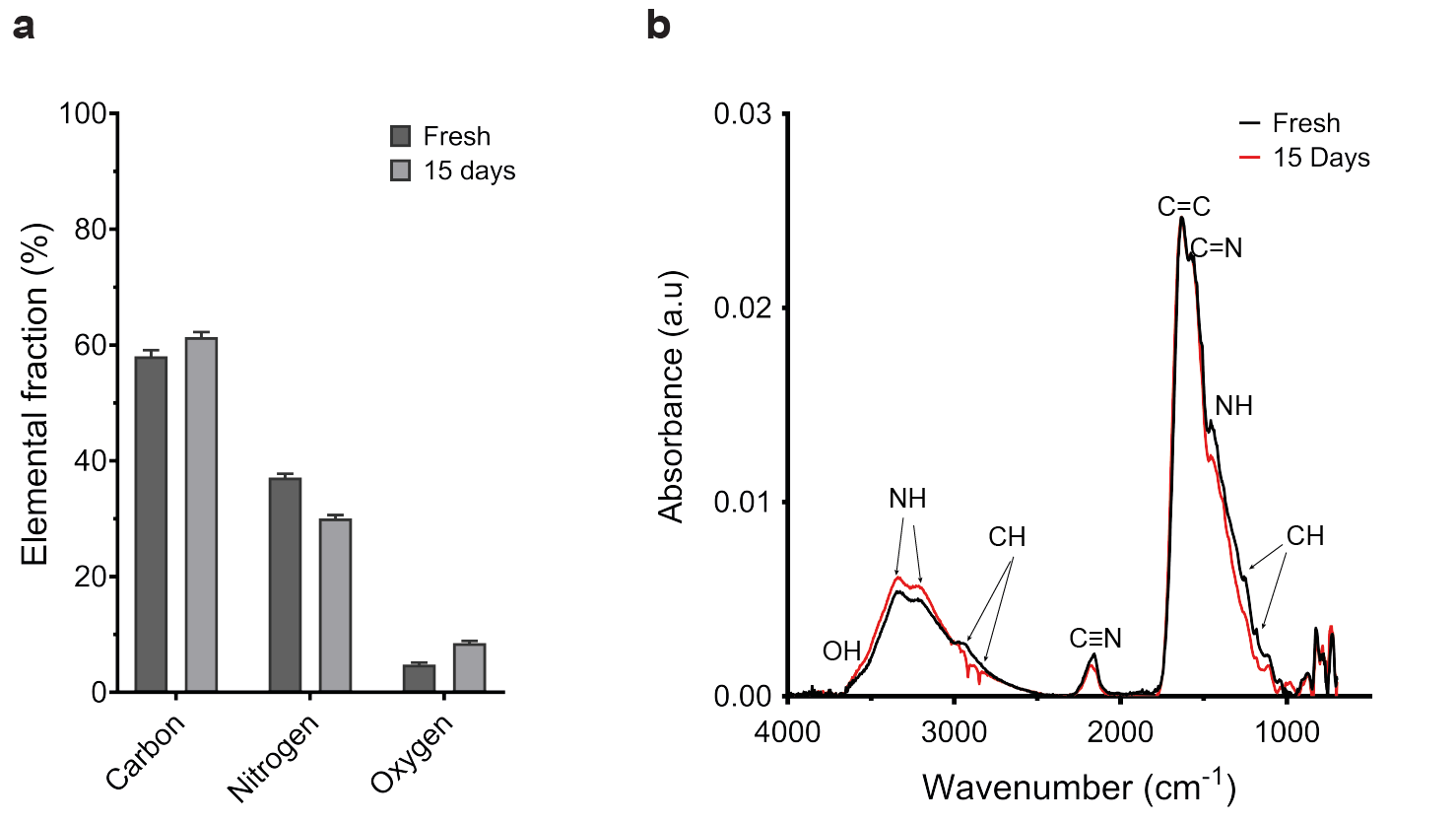
**

**Figure S1:** Chemical characterization of plasma polymerized nanoparticles using X-ray photoelectron spectroscopy (XPS) and Fourier transform infrared spectroscopy (FTIR). a) XPS measurements show that the elemental relative atomic fraction of carbon and nitrogen in freshly made nanoparticles (following exposure to atmosphere for 15 minutes) was 58% and 37 % respectively. Oxygen accounts for a relative elemental fraction of 5 % due to surface oxidation. Restructuring of the surface chemistry progresses over time, increasing the relative elemental fraction of oxygen and carbon to 9 % and 61% respectively while decreasing nitrogen to 30 %. b) FTIR further showed that amine and carboxyl functional moieties decorate the surface of PPN.


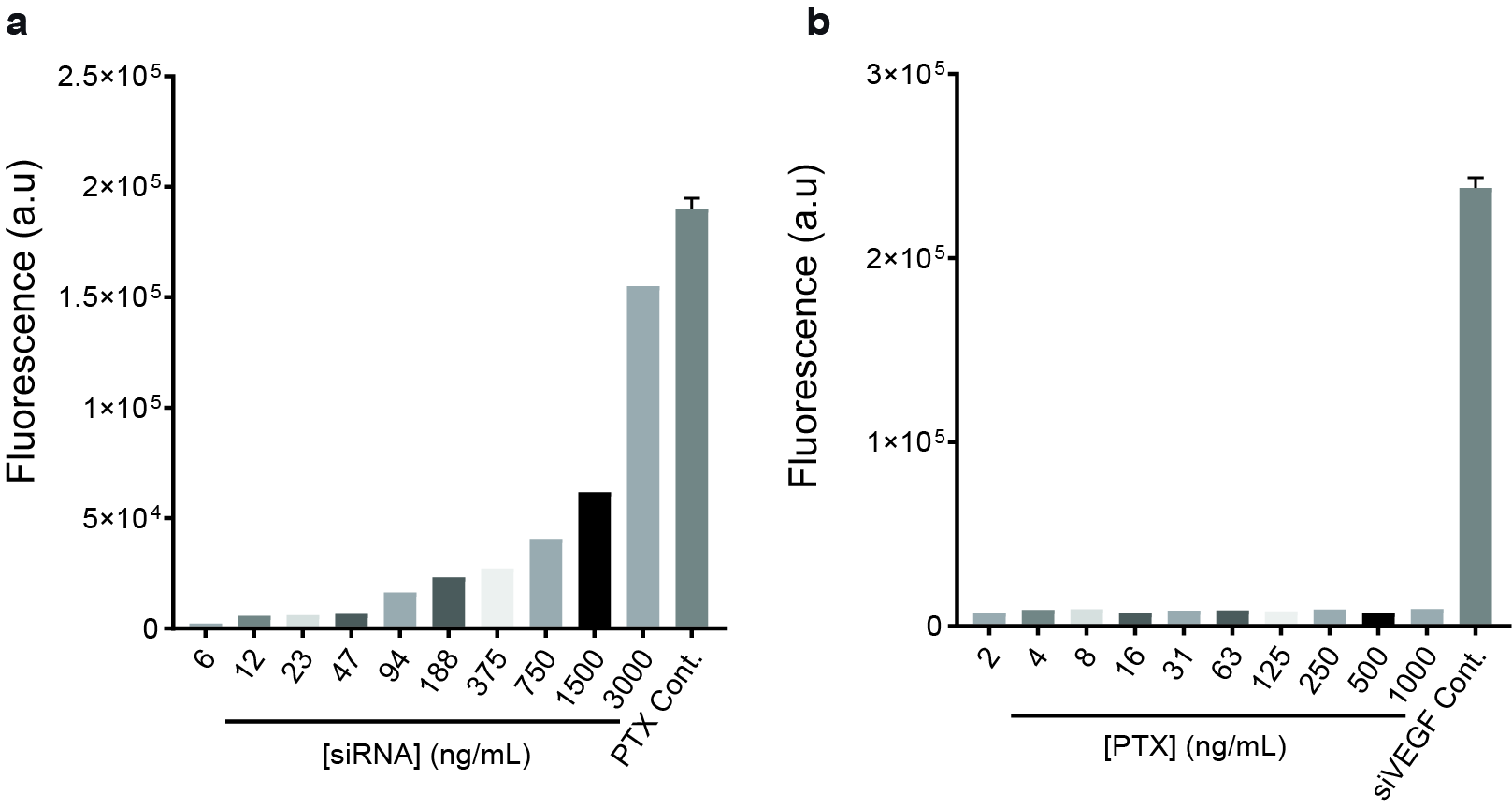


**Figure S2:** Molecular cargo elution from PPN surface upon sequential binding. a) Fluorescence intensity of PTX-OregonGree-488 in solution upon incubation of PPN-PTX with siVEGF solutions of various concentrations. PTX was partially desorbed from the surface of PPN at higher siVEGF concentrations. b) Fluorescence intensity of siVEGF(Cy5) in solution upon incubation of PPN-siVEGF(Cy5) with PTX solutions of various concentrations. No siVEGF was eluted from the PPN surface. All dual PPN formulations used in this work were prepared by sequential binding of siVEGF followed by PTX.

**
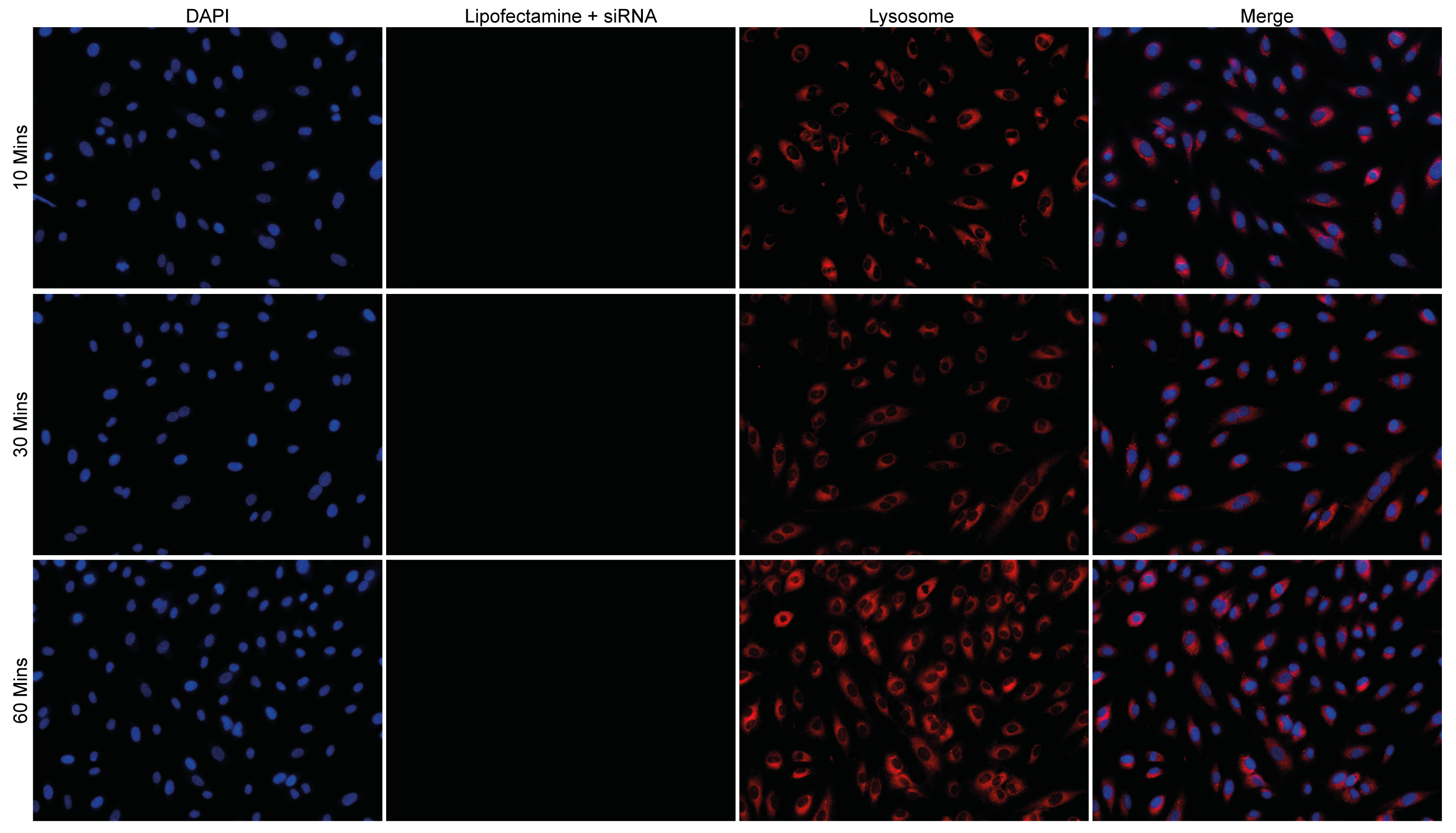
**

**Figure S3**. Confocal microscopy images of intracellular trafficking of Lipofectamine-siVEGF (Cy-5) suggests that internalization occurs significantly slower than PPN-siVEGF (Cy5).

**
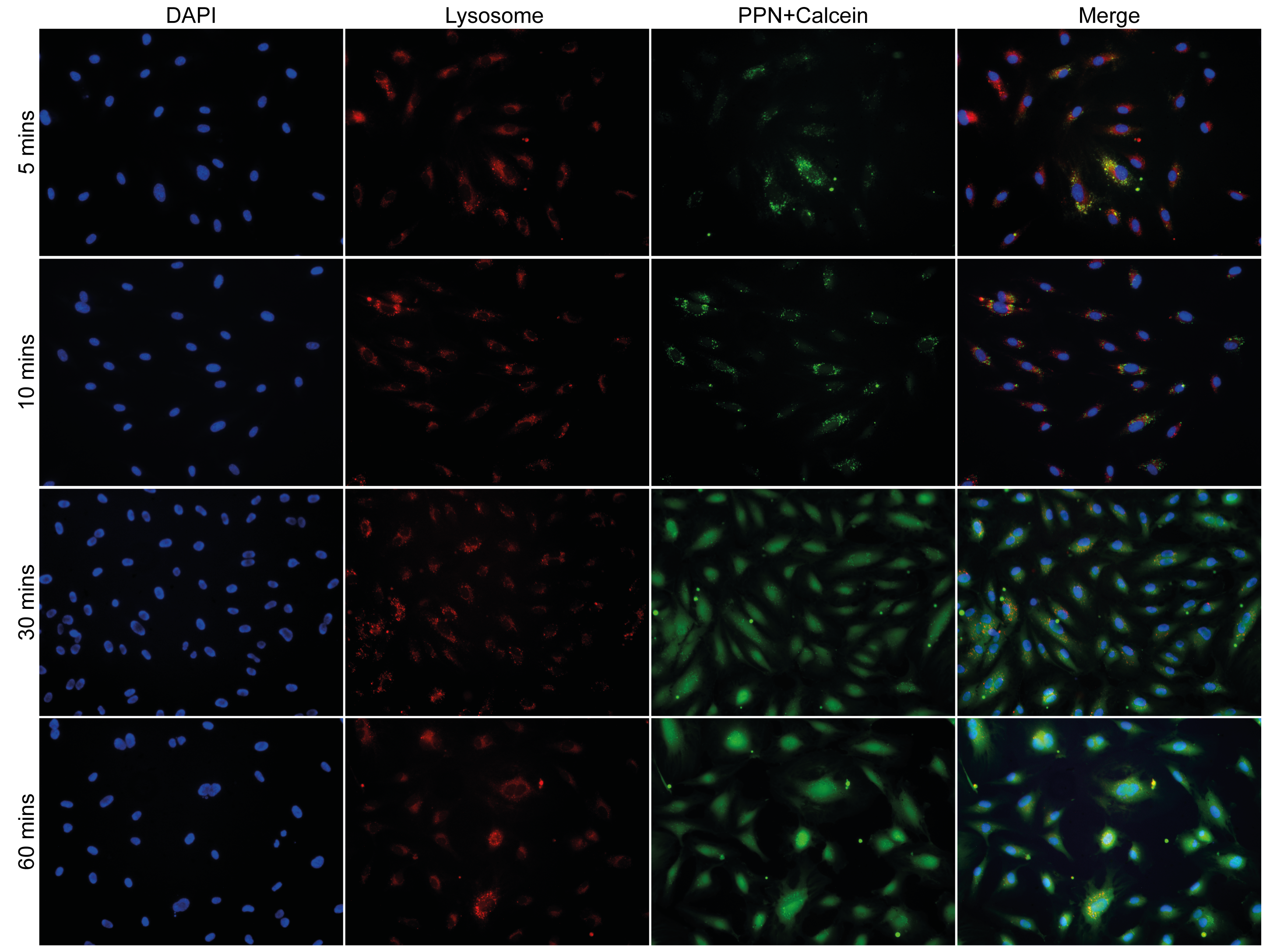
**

**Figure S4**. Confocal microscopy images of intracellular trafficking of Calcein bound to PPN (PPN-Calcein) in HCAECs. Punctate, low intensity and localised green fluorescence suggests that PPN-Calcein concentrate inside endosomes/lysosomes within the first 10 minutes following incubation with cells. Diffuse and intense green fluorescence is then observed after 30 minutes possibly due to membrane disruption and endosomal escape.


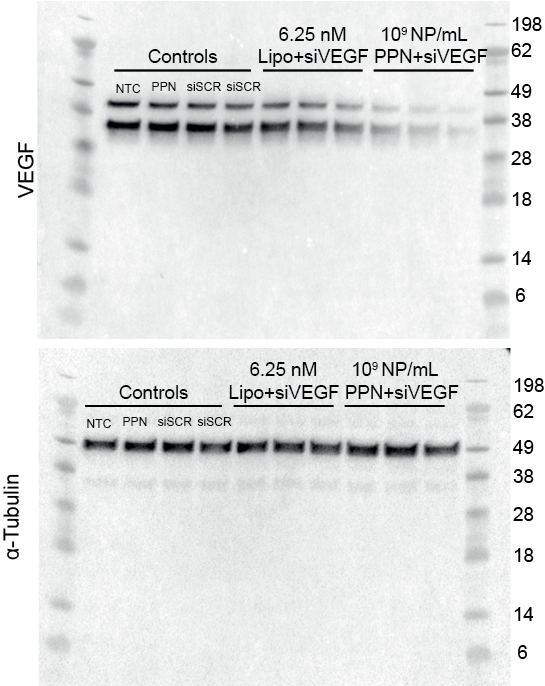


**Figure S5:** Representative western blot of VEGF expression in hCAECs cells upon incubation with PPN, PPN-siSCR, PPN-siVEGF, Lipo-siSCR and Lipo-siVEGF.


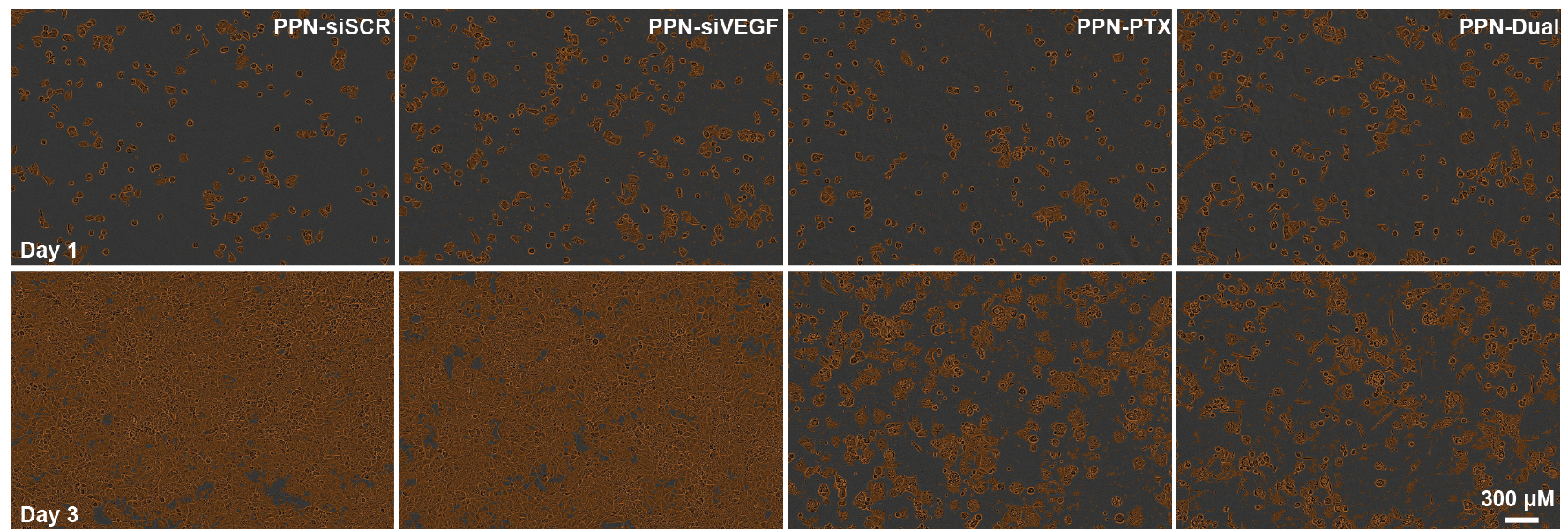


**Figure S6:** Representative images of MCF7 demonstrated striking differences in cell density and morphology 1- and 3-days following treatments, particularly with the PPN-PTX and PPN-Dual groups. (scale bar = 300 μm).


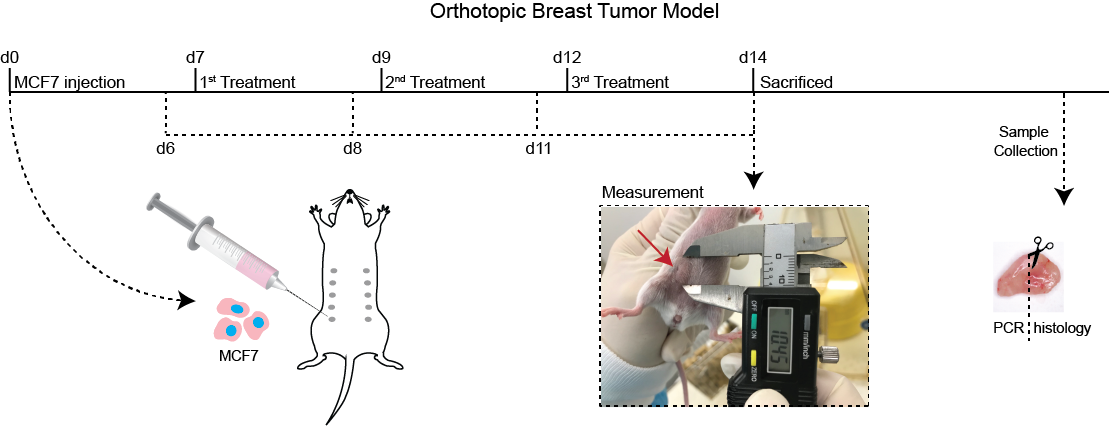


**Figure S7:** In-vivo evaluation of plasma polymerized nanoparticle (PPN) formulations *in vivo.* Schematic illustration of an established orthotopic breast tumor model used in this work to evaluate the therapeutic potential of siRNA and drug functionalized PPN.

**
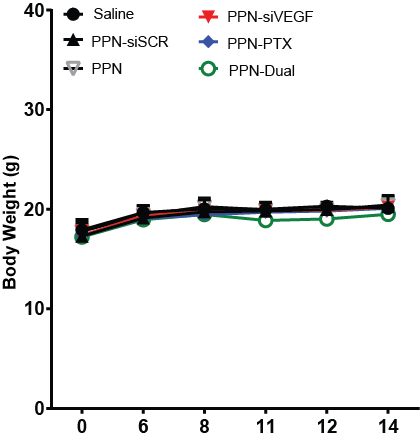
**

**Figure S8:** Mice body weight over the span of the treatment. Body weight of animals receiving PPN formulations did not change significantly compared to vehicle control.

**
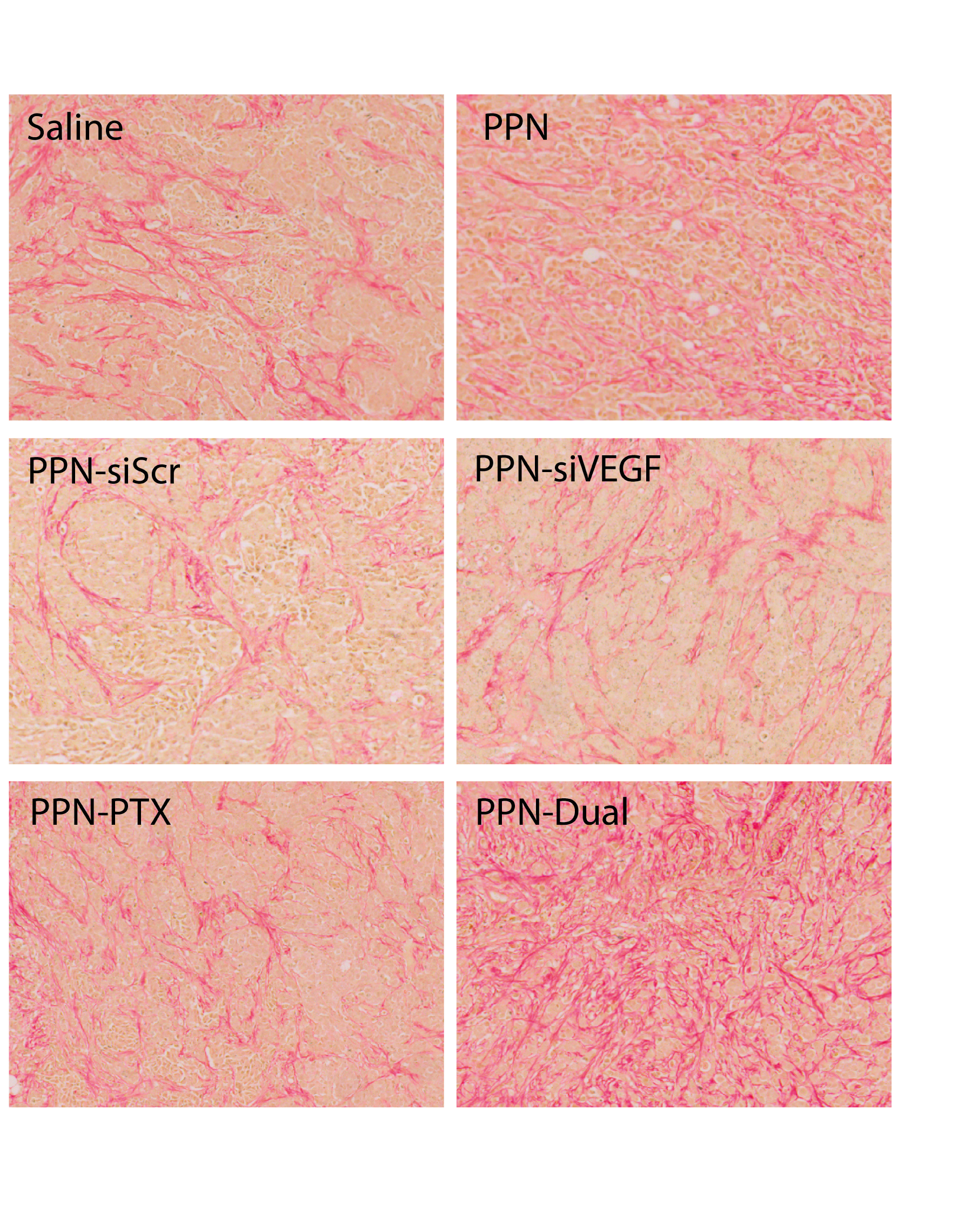
**

**Figure S9:** Represented images of tumors stained with picrosirius red for collagen deposition analysis.

**REFERENCES**

1. Santos, M. et al. Substrate geometry modulates self-assembly and collection of plasma polymerized nanoparticles. *Communications Physics* **2**, 52 (2019).

2. Santos, M. et al. Plasma Synthesis of Carbon-Based Nanocarriers for Linker-Free Immobilization of Bioactive Cargo. *ACS Applied Nano Materials* **1**, 580-594 (2018).
